# Development and pilot study of “Smart Cancer Care”: a platform for managing side effects of chemotherapy

**DOI:** 10.1186/s12913-023-09871-0

**Published:** 2023-08-29

**Authors:** Cheolkyung Sin, Hyeyeong Kim, Hyeon-Su Im, Minsu Ock, Su-Jin Koh

**Affiliations:** 1 Department of Hematology and Oncology, Ulsan University Hospital, University of Ulsan College of Medicine, Ulsan, Republic of Korea; 2 Department of Preventive Medicine, Ulsan University Hospital, University of Ulsan College of Medicine, Ulsan, Republic of Korea; 3 Department of Preventive Medicine, University of Ulsan College of Medicine, Seoul, Republic of Korea

**Keywords:** Drug therapy, Mobile Applications, Neoplasms, Drug-related side Effects and adverse reactions

## Abstract

**Background:**

As outpatient chemotherapy treatment increases, cancer patients receiving chemotherapy spend more time at home. In addition, since the types of chemotherapy are gradually expanding, it will be essential to prepare patient self-management strategies for various chemotherapy-related side effects. This study aimed to develop a platform (called Smart Cancer Care) to implement a chemotherapy side effect management program and to evaluate its feasibility.

**Methods:**

Smart Cancer Care comprises an application for patients and a dashboard for medical staff. Thirty-two symptoms to be managed using Smart Cancer Care were summarized through a literature review and Delphi. Management guidelines were developed based on the severity of each symptom (3 stages), and installed in Smart Cancer Care according to the Common Terminology Criteria for Adverse Events (CTCAE) v5.0 guidelines. To evaluate the feasibility of the developed application and medical dashboard, cancer patients and cancer treatment medical staff used Smart Cancer Care for 2 to 3 weeks and subsequently reported the experience of using them.

**Results:**

The patient application provided a list of symptoms according to the cancer type and anticancer drug enabling presence and severity of each symptom to be evaluated. Patients received management guidelines for symptoms based on the symptom evaluation results. On the medical staff dashboard, administrators and authorized medical personnel could access and assess information regarding side effects and symptom severity submitted by the patient. The feasibility and usefulness of Smart Cancer Care were confirmed through a pilot test targeting 30 patients and 24 chemotherapy-related medical staff. For patients, the evaluation score for the “The program will be helpful when seeing medical staff” item was the highest. For medical staff, the score for the “By checking the patient’s symptoms using the program, it helps to take appropriate measures for the patient” item was the highest. Although minor corrections were raised, most patients and medical staff expected that Smart Cancer Care would help their treatment.

**Conclusions:**

The configuration of the application and dashboard of Smart Cancer Care detailed in this study could be used for the development of a widely accepted platform to implement a chemotherapy side effect management program.

**Supplementary Information:**

The online version contains supplementary material available at 10.1186/s12913-023-09871-0.

## Background

Cancer patients experience an average of 14 symptoms caused by cancer and side effects of chemotherapy, which lead to difficulties in activities of daily life [1]. Since patients spend most of their time at home following chemotherapy, it is essential to evaluate and manage side effects and symptoms at home [1, 2]. Patients who received chemotherapy were about 20%p more likely to visit the emergency room and be hospitalized due to side effects compared to patients who did not [3]. Another previous study revealed that cancer patients who visited the emergency room due to side effects of cancer treatment, visited the emergency room on average 9.5 days after administration of the anticancer drug, and 72.8% of the patients who visited the emergency room were admitted for treatment, of which 6.5% died [4]. Therefore, it is crucial to detect and manage side effects in cancer patients in a timely manner.

The frequency of reported symptoms is higher when patients self-evaluate side effects due to cancer treatment compared to that of clinician evaluation [5]. Therefore, because symptom evaluation by medical staff is underestimated, it may be more appropriate to use patient-reported outcomes (PRO), using standardized questionnaires to evaluate symptoms accurately, whereby patients directly evaluate their health conditions without interpretation by medical staff or a third party [5]. Previous studies revealed that using PRO in patients undergoing cancer treatment aids in early symptom detection [6–9]. Specifically, monitoring symptoms to determine changes over time, improves quality of life, and enhances patient satisfaction by facilitating communication between patients and medical staff. In particular, using electronic PRO to treat cancer patients has been useful as the risk of COVID-19 infection led to restrictions on the use of medical institutions [10].

This study aimed to introduce “Smart Cancer Care,” a platform established to implement a management program for side effects due to chemotherapy in Republic of Korea (hereinafter Korea). Specifically, we presented how the patient application and the dashboard for medical staff, the two elements that make up Smart Cancer Care, were developed and configured, and how PRO was applied to them. In addition, the developed Smart Cancer Care was tested for cancer patients and medical staff (physicians and nurses) to evaluate its availability and usefulness.

## Methods

### Setting and participants

Smart Cancer Care targeted gastric, colorectal, lung, and breast cancers, which are common in Korea. In addition, anticancer drugs were classified into cytotoxic chemotherapy, targeted therapy, immunotherapy, and endocrine therapy.

### Monitoring symptoms and management guidelines

First, we reviewed the literature introducing tools (electronic PRO) for measuring the main symptoms that may occur during cancer treatment [11]. Symptoms (adverse drug events) that may occur in more than 1% of cancer patients when anticancer drugs are administered were then summarized. By combining these, the symptoms to be monitored for each anticancer drug were listed. The priority of symptoms to be monitored was selected using Delphi and an anticancer treatment expert. More details about Delphi are described in Supplemental file 1.

Based on the Delphi results and the existing literature review, a total of 32 symptoms to be managed in Smart Cancer Care were summarized. Existing literature reviewed for the development of management guidelines for side effects due to anticancer drugs are as follows: (1) Side Effects of Cancer Treatment (National Cancer Institute) [12], (2) ESMO Clinical Practice Guidelines: Supportive and Palliative Care [13], (3) Supportive Care; NCCN Clinical Practice Guidelines in Oncology (NCCN Guidelines®) [14]. The symptom stages were divided into stage 1 (preventive management), stage 2 (self-care), and stage 3 (consultation with medical staff or medical institution visit) after consulting previous studies; the guidelines were developed accordingly [15, 16].

Subsequently, the guidelines were revised and supplemented with the advice of experts (a total of 36) who were recommended by the palliative care division of the Korean Cancer Study Group and the Korean Oncology Nursing Society.

### Platform development and evaluation of its feasibility and usefulness

The Smart Cancer Care platform is largely divided into two parts: First, an application for patients who can self-evaluate symptoms according to the type of cancer and administered anticancer drugs and refer to management guidelines according to the results of the evaluation. Second, a dashboard for medical staff that can check the results entered by patients in real time. In order to evaluate the availability of the developed application for patients and the dashboard for medical staff, Smart Cancer Care was used for 2 to 3 weeks by cancer patients and medical staff involved in cancer treatment. Their experience using the platform was investigated. Referring to the feasibility survey items used in the previous study that attempted to evaluate the quality of life of cancer survivors using a wireless touch screen tablet personal computer [17], a survey questionnaire was developed to quantitatively evaluate the feasibility and usefulness of Smart Cancer Care regarding cognitive, psychological, written, and social aspects (a total of 12 items). The questionnaire also included open-ended questions to collect suggestions for improving Smart Cancer Care. The feasibility and usefulness was evaluated on a 5-point scale (0: Strongly disagree, 1: Disagree, 2: Neither agree nor disagree, 3: Agree, 4: Strongly agree); higher scores indicate greater usefulness, and negative questions were reverse-scored. The full questionnaire for patients and medical staff is attached as Supplemental file 2.

## Results

### Review of symptom monitoring and management guidelines

The 32 major side effects due to chemotherapy to be managed in Smart Cancer Care and the corresponding management guidelines are presented in Supplemental file 3.

### Application for patients

**Fig. 1:**
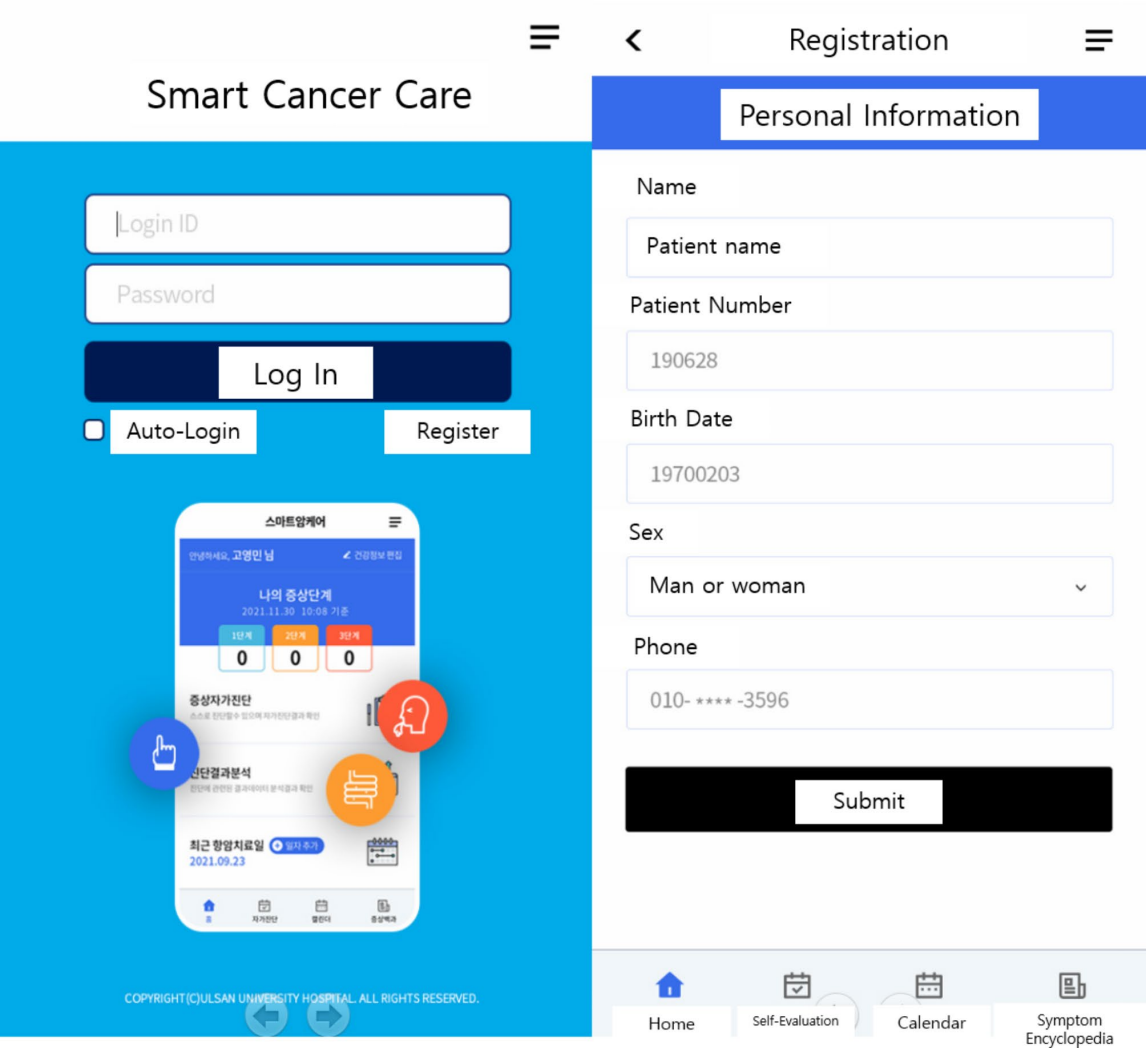
Log in and sign up for the Smart Cancer Care application

**Fig. 2:**
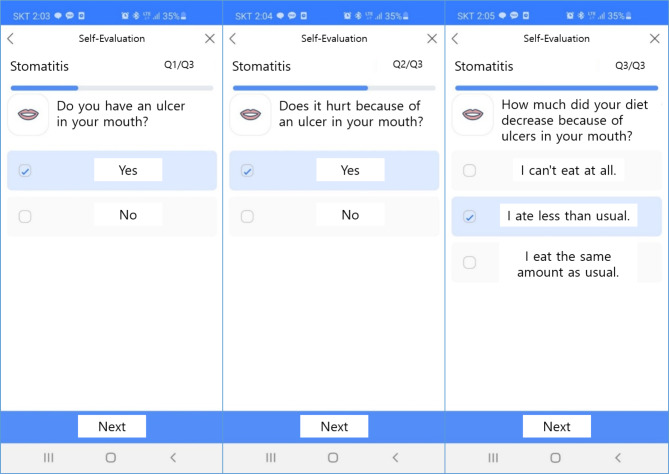
Example of evaluation of the presence and severity of symptom

**Fig. 3:**
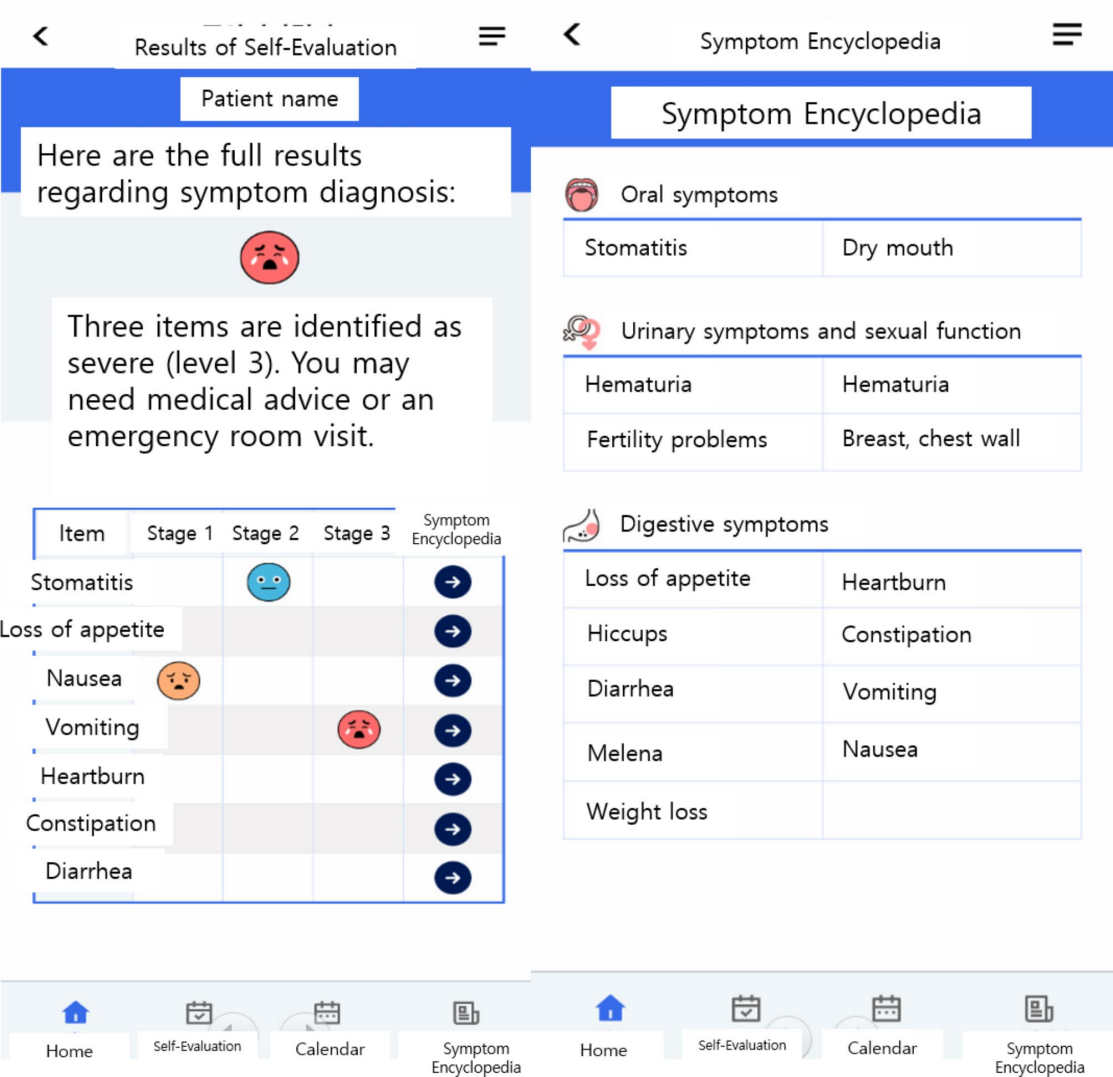
Symptom evaluation results and Symptom Encyclopedia

The application for patients can be obtained by downloading “Smart Cancer Care” from the Play Store or App Store. Once the application is installed, patients can sign up as members by entering their name, patient number, date of birth, sex and contact information (Fig. 1). Subsequently, they could self-evaluate their symptoms by entering medical information such as medical institution, diagnosis, type of anticancer drug, date of cancer treatment, and attending physician. Patients are provided with a list of symptoms to be evaluated according to the cancer type and the anticancer drugs, and allowed to evaluate the presence and severity of each symptom (Fig. 2). According to the symptom evaluation results, patients are provided with management guidelines for symptoms, which are classified into three stages as follows: Preventive management (stage 1), self-care (stage 2), and consultation with medical staff or medical institution visit (stage 3). Upon completion of all symptom evaluations, the overall symptom diagnosis results are presented. Full information on the side effects due to chemotherapy is available in a separate menu called Symptom Encyclopedia (Fig. 3).

### Dashboard for medical staff

**Fig. 4:**
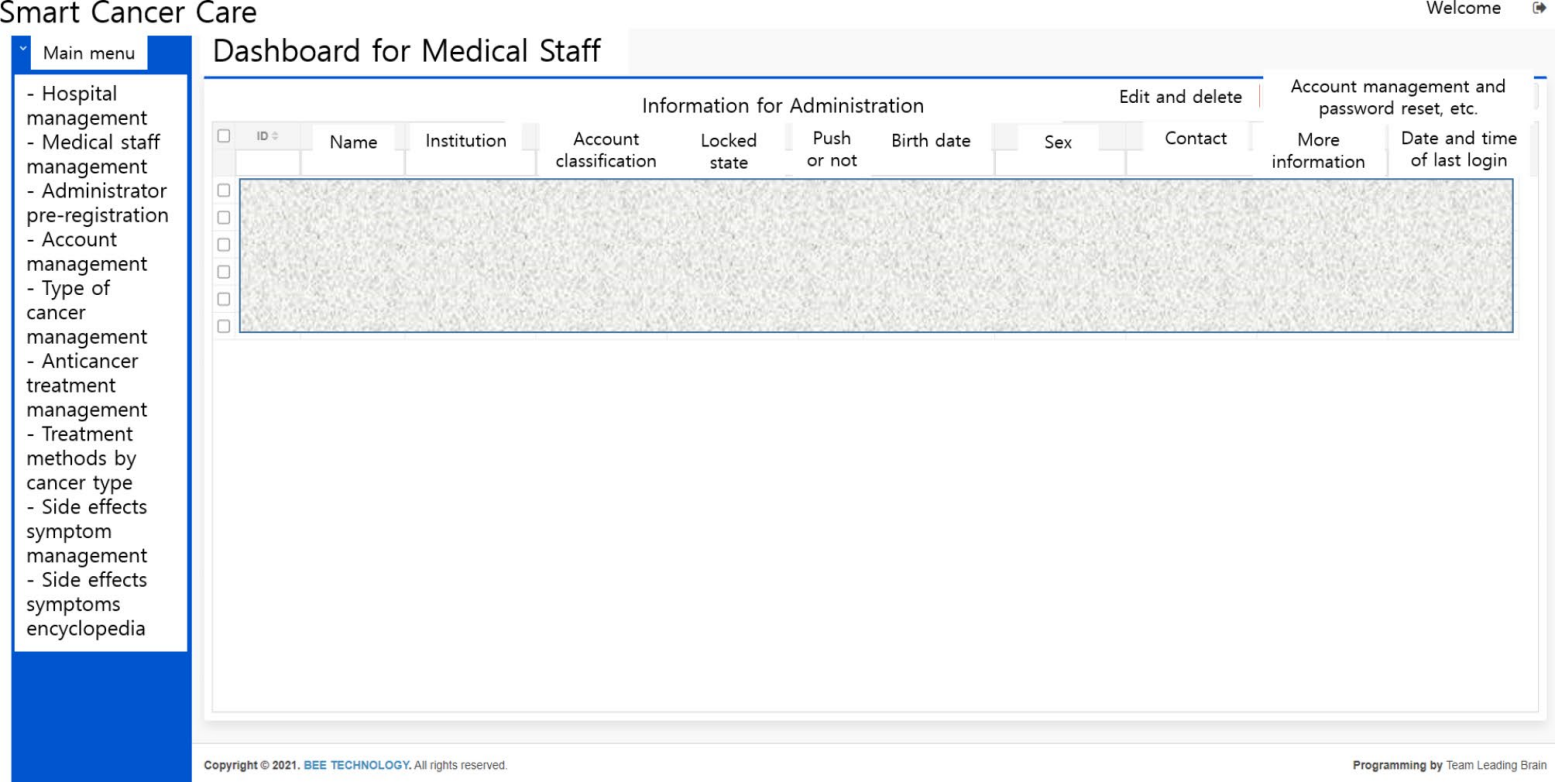
Administration mode in the medical staff dashboard

**Fig. 5:**
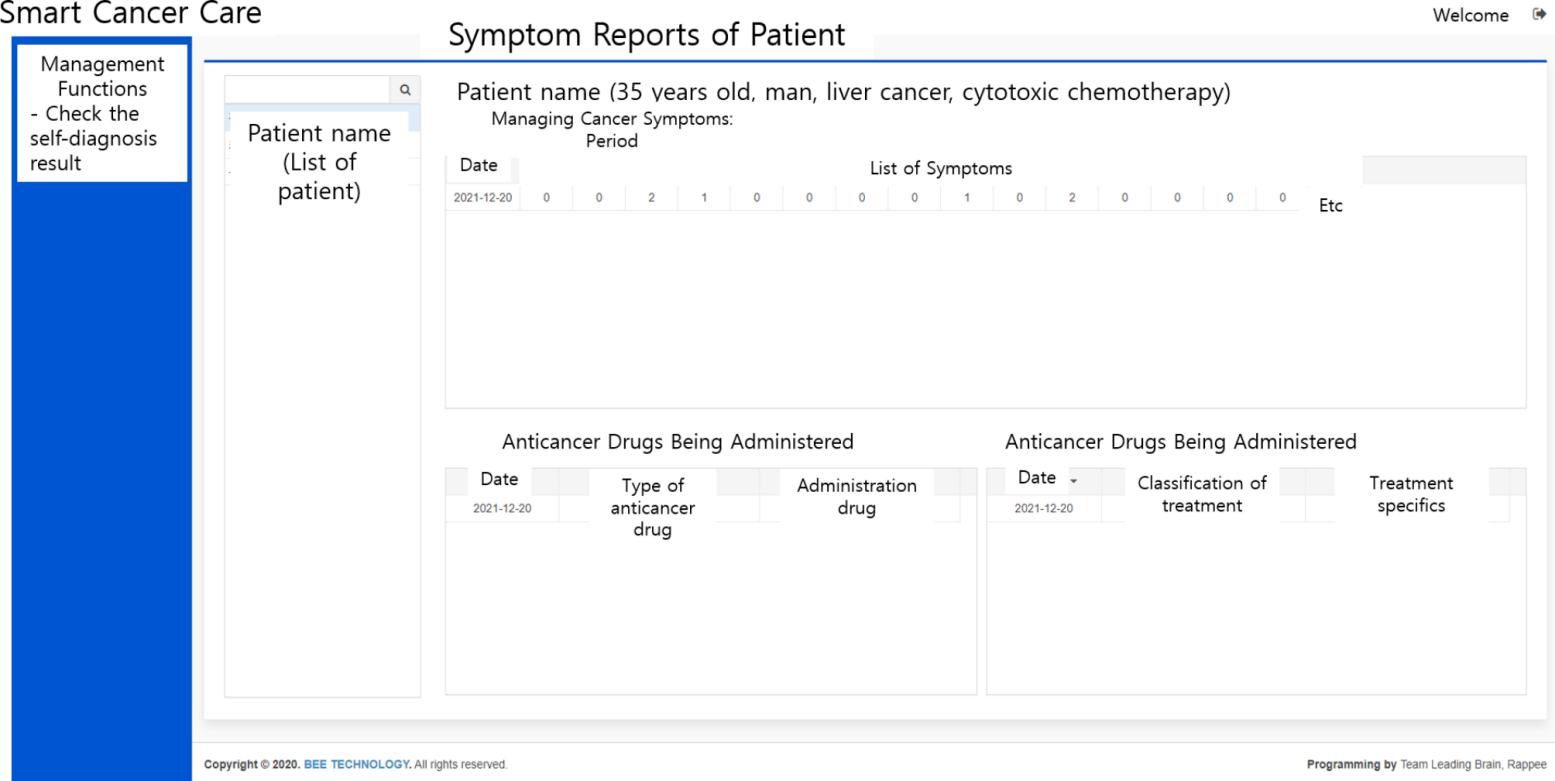
Patient information in the medical staff dashboard

The dashboard for medical staff enables administrators and authorized medical staff to assess the side effects and their severity entered by the patient. In particular, the administrator can adjust the authority of medical institutions and staff and set up another administrator (Fig. 4). The administrator can also set the patient’s cancer type and treatment method accordingly and modify the monitored symptoms and management guidelines. The medical staff can check the status of the symptom reports entered by the patients they treat, the anticancer drugs being administered, and the dates of chemotherapy administration (Fig. 5). In particular, nurses specializing in education can monitor this, encourage the use of Smart Cancer Care, and conduct consultations with patients to meet the various needs of patients. In addition, physicians can more accurately grasp the symptoms that patients complain of during outpatient treatment, helping them to form rapport with patients.

### Evaluation of program feasibility and usefulness

A survey was conducted including 30 patients and 24 medical staff involved in cancer treatment to evaluate the feasibility and usefulness of the program. The demographic and sociological characteristics of the participants are listed in Supplemental Tables 1 and 2. The overall mean (standard deviation) scores for the feasibility evaluation in patients and medical staff were 4.06 (0.50) and 3.99 (0.42), respectively (Table 1). For patients, the evaluation score for the “The program will be helpful when seeing medical staff” item was the highest (4.80), and the scores for the items, “I would recommend using this program for cancer patients around me” (4.77) and “The program was generally easy to use” (4.38), were also high. For medical staff, the score for the “By checking the patient’s symptoms using the program, it helps to take appropriate measures for the patient” item was the highest (4.89), and the scores for the items, “The program will help care for patients” (4.27), and “I would feel comfortable if the patients use the program to manage their side effect symptoms” (4.25), were also high.

**Table 1:** Patient and medical staff evaluations of platform

Evaluation area	Patient (n = 30)		Medical staff (n = 24)	
	Item	Mean ± Standard Deviation (Score range: 0–4)	Item	Mean ± Standard Deviation (Score range: 0–4)
Cognitive	I was able to understand the purpose of the program appropriately.	3.97 ± 1.02	I was able to understand the purpose of the program appropriately.	4.21 ± 0.50
	Overall, it was difficult to understand the contents of the program (reverse question).	3.73 ± 1.06	Overall, it was difficult to understand the contents of the program (reverse question).	3.21 ± 1.12
	The program was generally easy to use.	4.38 ± 1.11	The program was generally easy to use.	3.92 ± 0.70
Psychological	I feel uncomfortable entering my treatment status into the program (reverse question).	4.03 ± 0.98	I feel uncomfortable to check patient-related information through the program (inverse question).	3.65 ± 0.94
	I would feel comfortable if I managed side effects using the program	4.27 ± 0.51	I would feel comfortable if the patients use the program to manage their side effect symptoms	4.25 ± 0.52
	I would recommend using this program for cancer patients around me.	4.77 ± 0.63	I would recommend using this program for the medical staff around me.	3.77 ± 0.61
Written	The contents entered in the program reflect my treatment status properly.	3.81 ± 0.77	Patient information available through the program is appropriate.	3.96 ± 0.66
	There are too many items to respond to in the program’s questionnaire.	3.46 ± 0.91	By checking the symptoms of the patient through the program, it helps to take appropriate measures for the patients.	4.89 ± 0.55
	I need help from someone else to use the program.	3.10 ± 1.08	Additional functions are required to use the program.	3.69 ± 0.83
Social	The use of a management program for side effect symptoms will reduce hospital visits.	4.31 ± 0.80	The program will reduce the overall burden of managing side effect symptoms on patients.	4.25 ± 0.57
	The program will be helpful when seeing medical staff.	4.80 ± 0.45	The program will be helpful to care for patients.	4.27 ± 0.43
	I hope the program will be widely used.	4.04 ± 0.53	I hope the program will be widely used.	3.81 ± 0.55

The higher the score, the greater the usefulness of each area (0–4 points; Likert scale).

The experience of using the program by patients and medical staff collected using open-ended questions is summarized in Table 2. Although patients generally evaluated the application as not difficult to use, some patients found installing and logging into the application difficult. Nevertheless, they expected the contents of the application to be helpful in treatment. However, there was an opinion that an alarm should be given so as not to forget to evaluate the symptoms. The majority of medical staff stated that the program was intuitive and would be useful for patient care. However, they envision difficulties in using the application with older patients, and it was suggested that a program manual would be useful.

**Table 2:** Positive and negative experiences of patients and medical staff using Smart Cancer Care

	Patients	Medical staff
Positive	⦁ It was easy to use. ⦁ I could proceed without difficulty if I read the text of the application carefully. ⦁ I could see my status. ⦁ It will help the medical staff to treat patients. ⦁ It would be nice if my side effects or changes were delivered to the medical staff and reflected.	⦁ The icons for each list are easy to recognize. ⦁ It is similar to the app I use often and is intuitive. ⦁ It will be helpful to see the patient’s condition change and side effects at a glance. ⦁ It will be helpful for patients in the early stages of starting cancer treatment. ⦁ From the patient’s point of view, it would have an effect on reducing emotional instability.
Negative	⦁ It was not easy to use. ⦁ Log in is not working properly. ⦁ The installation process was a little complicated. ⦁ There are cases when I forget to do. ⦁ It is hard to do it alone. ⦁ It is easy to use but too simple.	⦁ I wish there were a user manual for the application and dashboard. ⦁ It is difficult for patients to enter personal information when they first start. ⦁ It is hard to log in. ⦁ From the nurse’s perspective, it does not seem necessary. ⦁ It seems complicated for older people to use.

## Discussion

In this study, we introduced Smart Cancer Care, an Information and Communication Technology (ICT)-based platform for managing side effects due to chemotherapy, and evaluated its feasibility and usefulness. Smart Cancer Care comprises an application for cancer patients that enables patients undergoing chemotherapy to self-evaluate side effects due to chemotherapy at home and receive guidelines on symptom management, and a dashboard for medical staff whereby they assess the side effects and their severity entered by the patient in real-time. Patients and medical staff found Smart Cancer Care useful in cognitive, psychological, written, and social aspects. Based on the results of this study that confirmed the feasibility of Smart Cancer Care, it is expected that Smart Cancer Care would be a vital tool in implementing a management program for symptoms due to chemotherapy in cancer patients.

This study is meaningful because it detailed the developmental process and screen configuration of an ICT-based platform to manage chemotherapy-related side effects. Several studies investigated the management of side effects due to chemotherapy using ICT-based platforms [15, 18–23], but few studies identified how these platforms were configured for the user’s convenience. Although there is potential for ICT-based management programs for chemotherapy side effects to improve cancer treatment; the program will only be successful when adoption and acceptance rates of ICT-based platforms are high [20, 24]. The configuration of the Smart Cancer Care application and dashboard detailed in this study can be used for developing a more widely accepted platform. Moreover, it is expected to be a reference for developing a symptom management platform after various surgeries, procedures, and radiation therapy, as well as chemotherapy.

Based on the experience of patients using Smart Cancer Care, it is believed that patients’ expectations for Smart Cancer Care were high, and they considered the application convenient. Specifically, cancer patients generally experience anxiety about the treatment itself [25, 26], but they anticipate that Smart Cancer Care can be useful because being able to communicate with medical staff and report side effects could help reduce anxiety about chemotherapy [19]. Improvements to Smart Cancer Care to increase convenience to patients were clearly identified. Simplifying the installation and login of Smart Cancer Care is also necessary. Since many cancer patients are older individuals, they are often not familiar with ICT; efforts to address this challenge should be continued. In addition, since ICT cannot replace all medical practices, having separate personnel to explain how to install and operate the application initially would be one way to overcome this problem.

The experience of medical staff using Smart Cancer Care was generally positive. It is known that when the information provided through ICT interferes with or aggravates existing medical services, acceptance of the technology declines [27]. Moreover, utilization of ICT-based platforms only increases when medical staff perceive them to be of practical assistance in medical treatment [28]. Smart Cancer Care obtained positive feedback on these two aspects; that is, it is expected to benefit patients while integrating with existing medical practices. However, medical staff believe that it would be difficult to utilize with older patients. It will be necessary to prepare and distribute user manuals for applications and dashboards in order to increase the convenience of using Smart Cancer Care.

To improve utilization of Smart Cancer Care by medical staff, it is necessary to consider linking the data collected from Smart Cancer Care with other medical records. From the medical staff’s perspective, linking the results regarding the reported chemotherapy-related side effects to the existing electronic medical records or clinical information system will make the data easier to review [16, 29]. This newly developed Smart Cancer Care has a limitation in that the data entered into the application can be identified through the dashboard linked to the existing medical records, but the contents of other medical records cannot be accessed through the dashboard. The challenge of integrating and managing PRO entered by patients at home with the information management system at the medical institution remains.

It is also significant that, to the authors knowledge, this study is the first attempt to utilize an ICT-based platform for the management of side effects of chemotherapy conducted in an Asian country. Several studies were previously conducted on managing chemotherapy-related side effects, but almost all were in Western countries [15, 18–23]. Considering that unmet medical care for cancer patients may differ depending on the sociocultural background [30], the possibility that cancer patients’ expectations and participation in management programs for side effects due to chemotherapy also vary based on sociocultural factors cannot be excluded [31]. Therefore, it is necessary to identify the points of improvement while using the newly developed Smart Cancer Care for patients with various socio-cultural backgrounds in the medical field.

A limitation of this study is that patients and medical staff who participated in the feasibility evaluation for Smart Cancer Care were recruited from a single medical institution located in Ulsan Metropolitan City, Korea. Therefore, the number of participants in this study is limited. To evaluate the acceptability of Smart Cancer Care, it is necessary to obtain feedback from medical staff and cancer patients at various medical institutions in further studies. In particular, a follow-up study will be needed on whether Smart Cancer Care can be used in patients who are not accustomed to handling electronic devices, such as the elderly. Furthermore, although Smart Cancer Care monitors 32 typical side effects that patients may experience from chemotherapy, the number of symptoms that patients may experience during chemotherapy may exceed 32. In the future, while using Smart Cancer Care in the field of care, it is necessary to check whether there are additional symptoms that need to be monitored within Smart Cancer Care.

## Conclusion

In this study, we developed Smart Cancer Care, an ICT-based platform for managing side effects due to chemotherapy, and evaluated its feasibility. Although more experience in using Smart Cancer Care may need to be accumulated, we determined that at least Smart Cancer Care would be feasible and useful through this study. As outpatient chemotherapy treatment increases, cancer patients receiving chemotherapy spend more time at home. In addition, since the types of chemotherapy are gradually expanding, it will be essential to prepare patient self-management strategies for various chemotherapy-related side effects. In the future, it is necessary to implement a management program for side effects due to chemotherapy in Korea using Smart Cancer Care while investing appropriate personnel and budget and evaluate its effectiveness. Furthermore, it is necessary to expand symptom management programs for patients undergoing cancer surgery as well as chemotherapy.

### Electronic supplementary material

Below is the link to the electronic supplementary material.

Supplementary Material 1

Supplementary Material 2

Supplementary Material 3

Supplementary Material 4

Supplementary Material 5

## Data Availability

All relevant data are within the paper and its supplementary information file.

## List of abbreviations

PRO: patient-reported outcomes
ICT: Information and Communication Technology
