# Supplementary material for: Development and pilot study of “Smart Cancer Care”: a platform for managing side effects of chemotherapy
Source: BMC Health Serv Res. 2023 Aug 29;23:922. doi: 10.1186/s12913-023-09871-0 (PMC10466749; doi:10.1186/s12913-023-09871-0)
Supplement: Supplementary file 1 — Supplementary Material 1 [file 12913_2023_9871_MOESM1_ESM.docx]

Supplemental table 1. Socio-demographic characteristics of survey participants for evaluation of program feasibility and usefulness (patients)

| Characteristics | | N (%) |
| --- | --- | --- |
| Age group | Under 40s | 5 (17.2) |
|  | 50s | 16 (55.2) |
|  | Over 60s | 8 (27.6) |
| Sex | Man | 10 (33.3) |
|  | Woman | 20 (66.7) |
| Education level | Less than middle school graduate | 5 (16.7) |
|  | High school graduate | 14 (46.7) |
|  | College graduate or higher | 11 (36.7) |
| Income level | No response | 1 (3.3) |
|  | Less than 1.5 million won per month | 7 (23.3) |
|  | More than 1.5 million won and less than 3 million won per month | 9 (30.0) |
|  | More than 3 million won and less than 5 million won per month | 9 (30.0) |
|  | Over 5 million won per month | 4 (13.3) |
| Current health status | Very bad | 3 (10.0) |
|  | Bad | 7 (23.3) |
|  | Moderate | 14 (46.7) |
|  | Good | 6 (20.0) |
|  | Very good | 0 (0.0) |
| Cancer treatment side effects symptom management | Do not know at all | 0 (0.0) |
|  | Do not know | 6 (20.0) |
|  | Know to some extent | 15 (50.0) |
|  | Know well | 9 (30.0) |
| Diagnosis | Head and neck cancer | 0 (0.0) |
|  | Lung cancer | 5 (16.7) |
|  | Gastric cancer | 3 (10.0) |
|  | Breast cancer | 14 (46.7) |
|  | Liver and biliary tract cancer | 0 (0.0) |
|  | Colorectal cancer | 8 (26.7) |
|  | Others | 0 (0.0) |
| Cancer stage | Do not know | 3 (10.0) |
|  | 1 stage | 4 (13.3) |
|  | 2 stage | 8 (26.7) |
|  | 3 stage | 8 (26.7) |
|  | 4 stage | 7 (23.3) |
