# Supplementary material for: Development and pilot study of “Smart Cancer Care”: a platform for managing side effects of chemotherapy
Source: BMC Health Serv Res. 2023 Aug 29;23:922. doi: 10.1186/s12913-023-09871-0 (PMC10466749; doi:10.1186/s12913-023-09871-0)
Supplement: Supplementary file 2 — Supplementary Material 2 [file 12913_2023_9871_MOESM2_ESM.docx]

Supplemental table 2. Socio-demographic characteristics of survey participants for evaluation of program feasibility and usefulness (medical staff)

| Characteristics | | N (%) |
| --- | --- | --- |
| Sex | Man | 4 (16.7) |
|  | Woman | 20 (83.3) |
| Occupation | General practitioner | 6 (25.0) |
|  | Resident | 2 (33.3) |
|  | Specialist | 4 (66.7) |
|  | Nurse | 18 (75.0) |
| Cancer treatment side effects symptom management | Do not know at all | 0 (0.0) |
|  | Do not know | 0 (0.0) |
|  | Know to some extent | 20 (83.3) |
|  | Know well | 4 (16.7) |
| Experience with cancer-related apps | Yes | 11 (45.8) |
|  | No | 13 (54.2) |
