# Supplementary material for: Development and pilot study of “Smart Cancer Care”: a platform for managing side effects of chemotherapy
Source: BMC Health Serv Res. 2023 Aug 29;23:922. doi: 10.1186/s12913-023-09871-0 (PMC10466749; doi:10.1186/s12913-023-09871-0)
Supplement: Supplementary file 3 — Supplementary Material 3 [file 12913_2023_9871_MOESM3_ESM.docx]

**Supplementary file 1: A Delphi survey to select major anticancer side effects for the purpose of developing a web-based program (Smart Cancer Care) for the evaluation and management of anticancer side effects in cancer patients**

∙ The specific process and results of the Delphi survey are being prepared for publication as a separate article. Here is an overview of the Delphi survey for the study, “Development and pilot study of “Smart Cancer Care”: a platform for managing side effects of chemotherapy”.

1. Criteria for selecting advisors who participated in the Delphi survey

∙ The advisory committee members were recommended by the Korean Cancer Study Group (<https://www.kcsg.org/en/index.do>) and the Korean Oncology Nursing Society (<https://www.kons.or.kr/main/main.php>).

∙ A total of 40 members participated in the Delphi survey by organizing 10 advisory committee members for each type of cancer (stomach cancer, colon cancer, lung cancer, breast cancer).

2. Delphi survey method

∙ The full Delphi survey, spanning three rounds of investigation, was conducted from September to December 2019.

∙Delphi questionnaire was developed for each type of cancer (stomach cancer, colorectal cancer, lung cancer, breast cancer), and the first round was conducted by e-mailing it to the advisory committee.

∙In order to induce re-agreement among the advisory members on the matters that were not agreed upon in the first round, the second round was conducted by sending the second round questionnaire to each advisory committee by e-mail.

∙In the case of the third round, 4 to 5 representative advisors were selected for each type of cancer, and a final agreement was reached through a face-to-face meeting.

3. Delphi questionnaire

∙ On the next page, an example of the Delphi First Round Survey for breast cancer is provided.

| **Delphi Survey (Breast Cancer) - Round 1 -** |
| --- |
| Thank you for participating as an expert breast cancer advisory committee member in the expert Delphi survey on the management of chemotherapy side effects.  Side effects of breast cancer-related chemotherapy are summarized by type of anticancer drug (1. Cytotxic chemotherapy / 2. Targeted therapy / 3. Endocrine therapy). We seek the opinions of expert advisors on the significance of symptoms, the urgency of intervention, and the usefulness of symptom management.  1) Significance of symptoms: how great and important the symptoms affect the health and life of cancer patients  2) Urgency of intervention: the degree to which intervention is necessary considering how quickly the problem needs to be resolved  3) Usefulness of symptom management: consider whether there is an effective way to manage symptoms and how much side effects can be improved and quality of life can be improved by symptom management  For the first Delphi survey, please fill out and send your responses by October 11th (Friday).  The second survey will be conducted at the end of October, and the third face-to-face meeting will be held at the Korean Society of Medical Oncology conference on November 7-8.  We promise that the results of the advisory members' responses will never be used for any purpose other than research, and we plan to pay a small consulting fee.  Thank you very much for participating in this study despite your busy schedule.  Thanks in advance. |

The criteria for summarizing the symptoms included in the Delphi questionnaire are as follows.

1) The main symptoms of breast cancer patients are included as follows. (Emens LA, Davidson NE. The follow-up of breast cancer. Semin Oncol 2003; 30:338)

- Locoregional recurrence: breast mass, chest wall mass, nipple discharge, rash, LN enlargement

- Systemic recurrence: bone pain, chest pain, cough, dyspnea, weight loss, anorexia, persistent headache

2) The regimen for each type of anticancer drug used for breast cancer was categorized into cytotoxic chemotherapy and targeted therapy, and reviewed by a representative advisory committee member for each cancer type. The combination of targeted therapy and cytotoxic chemotherapy is classified as targeted therapy.

| **Cytotoxic chemotherapy** | **Targeted agent** | **Endocrine therapy** |
| --- | --- | --- |
| AC (Doxorubicin/Cyclophosphamide)  ACD(Doxorubicin/Cyclophosphamide/Dextrazoxane)  FAC (5-FU/Doxorubicin/Cyclophosphamide)  FEC (5-FU/ Epirubicin/ Cyclophosphamide)  Docetaxel  Paclitaxel, Weekly – Paclitaxel  Albumin- bound paclitaxel (Nab-Paclitaxel)  TC (Docetaxel/ Cyclophosphamide)  TAC (Docetaxel/Doxorubicin/Cyclophosphamide)  AD (Doxorubicin/ Docetaxel)  AT (Doxorubicin/Paclitaxel)  Paclitaxel/ Cisplatin  Paclitaxel/ Carboplatin  Capecitabine  DX (Docetaxel/ Capecitabine)  Capecitabine/Vinorelbine  GN (Gemcitabine/Vinorelbine)  GP (Gemcitabine/Cisplatin)  Vinorelbine  Eribulin  CMF (Cyclophosphamide/Methotrexate/5-FU)  ICE (lfosfamide/Carboplatin/Etoposide) | Trastuzumab SC or IV  Docetaxel/ Trastuzumab SC or IV  Paclitaxel/ Trastuzumab SC or IV  TCH (Docetaxel/ Carboplatin/ Trastuzumab SC or IV)  Trastuzumab/ Pertuzumab  Docetaxel/ Trastuzumab/ Pertuzumab  Paclitaxel/ Trastuzumab/ Pertuzumab  TCHP (Docetaxel/ Carboplatin/ Trastuzumab SC or IV/ Pertuzumab)  Anastrozole/Trastuzumab  Lapatinib/Letrozole  Lapatinib/ Capecitabine  Trastuzumab emtansin ? | Tamoxifen  Anastrozole  Letrozole  Fulvestrant  Letrozole/Palbociclib  Palbociclib/Fulvestrant  Exmestane  Everolimus/Exmestane |

3) The side effects of anti-cancer regimen were summarized by referring to the pivotal trial, and reference literature was selected by referring to the NCCN guideline and UpToDate.

4) Based on the adverse events data in the literature (including original article and supplementary), all side effects with an incidence of 1% or more were investigated, and only non-hematologic toxicities with an incidence of 5% or more were included in the Delphi questionnaire.

5) Terminology was based on the side effects of CTCAE version 5.0, and terms from the Korean version of PRO-CTCAE were used for Korean. If the Korean term is not in the Korean version, it is translated based on the medical terminology.

6) Symptoms and side effects are listed by system.

7) Immunotherapy was not included in this survey because it was planned to be investigated separately by expert Delphi.

1. Cytotoxic chemotherapy

| 1) Significance of symptoms  (How great and important the effect of symptoms on the health status and life of cancer patients) | Not at all | | | | | |  | | Very much | | | |
| --- | --- | --- | --- | --- | --- | --- | --- | --- | --- | --- | --- | --- |
|  |  | | | | | | | | | | | |
|  | 1 | 2 | 3 | 4 | 5 | 6 | | 7 | | 8 | 9 |  |
| Oral/Pharyngeal mucositis |  |  |  |  |  |  | |  | |  |  |  |
|  |  |  |  |  |  |  | |  | |  |  |  |
| Anorexia |  |  |  |  |  |  | |  | |  |  |  |
|  |  |  |  |  |  |  | |  | |  |  |  |
| Weight loss |  |  |  |  |  |  | |  | |  |  |  |
|  |  |  |  |  |  |  | |  | |  |  |  |
| Nausea |  |  |  |  |  |  | |  | |  |  |  |
|  |  |  |  |  |  |  | |  | |  |  |  |
| Vomiting |  |  |  |  |  |  | |  | |  |  |  |
|  |  |  |  |  |  |  | |  | |  |  |  |
| Heartburn |  |  |  |  |  |  | |  | |  |  |  |
|  |  |  |  |  |  |  | |  | |  |  |  |
| Constipation |  |  |  |  |  |  | |  | |  |  |  |
|  |  |  |  |  |  |  | |  | |  |  |  |
| Diarrhea |  |  |  |  |  |  | |  | |  |  |  |
|  |  |  |  |  |  |  | |  | |  |  |  |
| Dyspnea |  |  |  |  |  |  | |  | |  |  |  |
|  |  |  |  |  |  |  | |  | |  |  |  |
| Edema limbs |  |  |  |  |  |  | |  | |  |  |  |
|  |  |  |  |  |  |  | |  | |  |  |  |
| Palpitation |  |  |  |  |  |  | |  | |  |  |  |
|  |  |  |  |  |  |  | |  | |  |  |  |
| Breast or chest wall mass |  |  |  |  |  |  | |  | |  |  |  |
|  |  |  |  |  |  |  | |  | |  |  |  |
| Lymph node enlargement |  |  |  |  |  |  | |  | |  |  |  |
|  |  |  |  |  |  |  | |  | |  |  |  |
| Rash |  |  |  |  |  |  | |  | |  |  |  |
|  |  |  |  |  |  |  | |  | |  |  |  |
| Hair loss |  |  |  |  |  |  | |  | |  |  |  |
|  |  |  |  |  |  |  | |  | |  |  |  |
| Hand and-Foot syndrome |  |  |  |  |  |  | |  | |  |  |  |
|  |  |  |  |  |  |  | |  | |  |  |  |
| Paronychia |  |  |  |  |  |  | |  | |  |  |  |
|  |  |  |  |  |  |  | |  | |  |  |  |
| Injection site reaction |  |  |  |  |  |  | |  | |  |  |  |
|  |  |  |  |  |  |  | |  | |  |  |  |
| Peripheral neuropathy |  |  |  |  |  |  | |  | |  |  |  |
|  |  |  |  |  |  |  | |  | |  |  |  |
| Dizziness |  |  |  |  |  |  | |  | |  |  |  |
|  |  |  |  |  |  |  | |  | |  |  |  |
| Watery eyes |  |  |  |  |  |  | |  | |  |  |  |
|  |  |  |  |  |  |  | |  | |  |  |  |
| Pain |  |  |  |  |  |  | |  | |  |  |  |
|  |  |  |  |  |  |  | |  | |  |  |  |
| Insomnia |  |  |  |  |  |  | |  | |  |  |  |
|  |  |  |  |  |  |  | |  | |  |  |  |
| Fatigue |  |  |  |  |  |  | |  | |  |  |  |
|  |  |  |  |  |  |  | |  | |  |  |  |
| Dysuria |  |  |  |  |  |  | |  | |  |  |  |
|  |  |  |  |  |  |  | |  | |  |  |  |
| Hot flashes |  |  |  |  |  |  | |  | |  |  |  |
|  |  |  |  |  |  |  | |  | |  |  |  |
| Fever |  |  |  |  |  |  | |  | |  |  |  |
|  |  |  |  |  |  |  | |  | |  |  |  |
| Bleeding |  |  |  |  |  |  | |  | |  |  |  |
|  |  |  |  |  |  |  | |  | |  |  |  |

| 2) Urgency of intervention  (The extent to which intervention is necessary, given how quickly the problem needs to be resolved) | Not at all | | | | | |  | | Very much | | | |
| --- | --- | --- | --- | --- | --- | --- | --- | --- | --- | --- | --- | --- |
|  |  | | | | | | | | | | | |
|  | 1 | 2 | 3 | 4 | 5 | 6 | | 7 | | 8 | 9 |  |
| Oral/Pharyngeal mucositis |  |  |  |  |  |  | |  | |  |  |  |
|  |  |  |  |  |  |  | |  | |  |  |  |
| Anorexia |  |  |  |  |  |  | |  | |  |  |  |
|  |  |  |  |  |  |  | |  | |  |  |  |
| Weight loss |  |  |  |  |  |  | |  | |  |  |  |
|  |  |  |  |  |  |  | |  | |  |  |  |
| Nausea |  |  |  |  |  |  | |  | |  |  |  |
|  |  |  |  |  |  |  | |  | |  |  |  |
| Vomiting |  |  |  |  |  |  | |  | |  |  |  |
|  |  |  |  |  |  |  | |  | |  |  |  |
| Heartburn |  |  |  |  |  |  | |  | |  |  |  |
|  |  |  |  |  |  |  | |  | |  |  |  |
| Constipation |  |  |  |  |  |  | |  | |  |  |  |
|  |  |  |  |  |  |  | |  | |  |  |  |
| Diarrhea |  |  |  |  |  |  | |  | |  |  |  |
|  |  |  |  |  |  |  | |  | |  |  |  |
| Dyspnea |  |  |  |  |  |  | |  | |  |  |  |
|  |  |  |  |  |  |  | |  | |  |  |  |
| Edema limbs |  |  |  |  |  |  | |  | |  |  |  |
|  |  |  |  |  |  |  | |  | |  |  |  |
| Palpitation |  |  |  |  |  |  | |  | |  |  |  |
|  |  |  |  |  |  |  | |  | |  |  |  |
| Breast or chest wall mass |  |  |  |  |  |  | |  | |  |  |  |
|  |  |  |  |  |  |  | |  | |  |  |  |
| Lymph node enlargement |  |  |  |  |  |  | |  | |  |  |  |
|  |  |  |  |  |  |  | |  | |  |  |  |
| Rash |  |  |  |  |  |  | |  | |  |  |  |
|  |  |  |  |  |  |  | |  | |  |  |  |
| Hair loss |  |  |  |  |  |  | |  | |  |  |  |
|  |  |  |  |  |  |  | |  | |  |  |  |
| Hand and-Foot syndrome |  |  |  |  |  |  | |  | |  |  |  |
|  |  |  |  |  |  |  | |  | |  |  |  |
| Paronychia |  |  |  |  |  |  | |  | |  |  |  |
|  |  |  |  |  |  |  | |  | |  |  |  |
| Injection site reaction |  |  |  |  |  |  | |  | |  |  |  |
|  |  |  |  |  |  |  | |  | |  |  |  |
| Peripheral neuropathy |  |  |  |  |  |  | |  | |  |  |  |
|  |  |  |  |  |  |  | |  | |  |  |  |
| Dizziness |  |  |  |  |  |  | |  | |  |  |  |
|  |  |  |  |  |  |  | |  | |  |  |  |
| Watery eyes |  |  |  |  |  |  | |  | |  |  |  |
|  |  |  |  |  |  |  | |  | |  |  |  |
| Pain |  |  |  |  |  |  | |  | |  |  |  |
|  |  |  |  |  |  |  | |  | |  |  |  |
| Insomnia |  |  |  |  |  |  | |  | |  |  |  |
|  |  |  |  |  |  |  | |  | |  |  |  |
| Fatigue |  |  |  |  |  |  | |  | |  |  |  |
|  |  |  |  |  |  |  | |  | |  |  |  |
| Dysuria |  |  |  |  |  |  | |  | |  |  |  |
|  |  |  |  |  |  |  | |  | |  |  |  |
| Hot flashes |  |  |  |  |  |  | |  | |  |  |  |
|  |  |  |  |  |  |  | |  | |  |  |  |
| Fever |  |  |  |  |  |  | |  | |  |  |  |
|  |  |  |  |  |  |  | |  | |  |  |  |
| Bleeding |  |  |  |  |  |  | |  | |  |  |  |
|  |  |  |  |  |  |  | |  | |  |  |  |

| 3) Usefulness of symptom management  (Consider whether there are effective ways to manage symptoms, and how much side effects can be improved and quality of life improved by symptom management) | Not at all | | | | | |  | | Very much | | | |
| --- | --- | --- | --- | --- | --- | --- | --- | --- | --- | --- | --- | --- |
|  |  | | | | | | | | | | | |
|  | 1 | 2 | 3 | 4 | 5 | 6 | | 7 | | 8 | 9 |  |
| Oral/Pharyngeal mucositis |  |  |  |  |  |  | |  | |  |  |  |
|  |  |  |  |  |  |  | |  | |  |  |  |
| Anorexia |  |  |  |  |  |  | |  | |  |  |  |
|  |  |  |  |  |  |  | |  | |  |  |  |
| Weight loss |  |  |  |  |  |  | |  | |  |  |  |
|  |  |  |  |  |  |  | |  | |  |  |  |
| Nausea |  |  |  |  |  |  | |  | |  |  |  |
|  |  |  |  |  |  |  | |  | |  |  |  |
| Vomiting |  |  |  |  |  |  | |  | |  |  |  |
|  |  |  |  |  |  |  | |  | |  |  |  |
| Heartburn |  |  |  |  |  |  | |  | |  |  |  |
|  |  |  |  |  |  |  | |  | |  |  |  |
| Constipation |  |  |  |  |  |  | |  | |  |  |  |
|  |  |  |  |  |  |  | |  | |  |  |  |
| Diarrhea |  |  |  |  |  |  | |  | |  |  |  |
|  |  |  |  |  |  |  | |  | |  |  |  |
| Dyspnea |  |  |  |  |  |  | |  | |  |  |  |
|  |  |  |  |  |  |  | |  | |  |  |  |
| Edema limbs |  |  |  |  |  |  | |  | |  |  |  |
|  |  |  |  |  |  |  | |  | |  |  |  |
| Palpitation |  |  |  |  |  |  | |  | |  |  |  |
|  |  |  |  |  |  |  | |  | |  |  |  |
| Breast or chest wall mass |  |  |  |  |  |  | |  | |  |  |  |
|  |  |  |  |  |  |  | |  | |  |  |  |
| Lymph node enlargement |  |  |  |  |  |  | |  | |  |  |  |
|  |  |  |  |  |  |  | |  | |  |  |  |
| Rash |  |  |  |  |  |  | |  | |  |  |  |
|  |  |  |  |  |  |  | |  | |  |  |  |
| Hair loss |  |  |  |  |  |  | |  | |  |  |  |
|  |  |  |  |  |  |  | |  | |  |  |  |
| Hand and-Foot syndrome |  |  |  |  |  |  | |  | |  |  |  |
|  |  |  |  |  |  |  | |  | |  |  |  |
| Paronychia |  |  |  |  |  |  | |  | |  |  |  |
|  |  |  |  |  |  |  | |  | |  |  |  |
| Injection site reaction |  |  |  |  |  |  | |  | |  |  |  |
|  |  |  |  |  |  |  | |  | |  |  |  |
| Peripheral neuropathy |  |  |  |  |  |  | |  | |  |  |  |
|  |  |  |  |  |  |  | |  | |  |  |  |
| Dizziness |  |  |  |  |  |  | |  | |  |  |  |
|  |  |  |  |  |  |  | |  | |  |  |  |
| Watery eyes |  |  |  |  |  |  | |  | |  |  |  |
|  |  |  |  |  |  |  | |  | |  |  |  |
| Pain |  |  |  |  |  |  | |  | |  |  |  |
|  |  |  |  |  |  |  | |  | |  |  |  |
| Insomnia |  |  |  |  |  |  | |  | |  |  |  |
|  |  |  |  |  |  |  | |  | |  |  |  |
| Fatigue |  |  |  |  |  |  | |  | |  |  |  |
|  |  |  |  |  |  |  | |  | |  |  |  |
| Dysuria |  |  |  |  |  |  | |  | |  |  |  |
|  |  |  |  |  |  |  | |  | |  |  |  |
| Hot flashes |  |  |  |  |  |  | |  | |  |  |  |
|  |  |  |  |  |  |  | |  | |  |  |  |
| Fever |  |  |  |  |  |  | |  | |  |  |  |
|  |  |  |  |  |  |  | |  | |  |  |  |
| Bleeding |  |  |  |  |  |  | |  | |  |  |  |
|  |  |  |  |  |  |  | |  | |  |  |  |

1. Targeted agent

| 1) Significance of symptoms  (How great and important the effect of symptoms on the health status and life of cancer patients) | Not at all | | | | | |  | | Very much | | | |
| --- | --- | --- | --- | --- | --- | --- | --- | --- | --- | --- | --- | --- |
|  |  | | | | | | | | | | | |
|  | 1 | 2 | 3 | 4 | 5 | 6 | | 7 | | 8 | 9 |  |
| Oral/Pharyngeal mucositis |  |  |  |  |  |  | |  | |  |  |  |
|  |  |  |  |  |  |  | |  | |  |  |  |
| Anorexia |  |  |  |  |  |  | |  | |  |  |  |
|  |  |  |  |  |  |  | |  | |  |  |  |
| Weight loss |  |  |  |  |  |  | |  | |  |  |  |
|  |  |  |  |  |  |  | |  | |  |  |  |
| Nausea |  |  |  |  |  |  | |  | |  |  |  |
|  |  |  |  |  |  |  | |  | |  |  |  |
| Vomiting |  |  |  |  |  |  | |  | |  |  |  |
|  |  |  |  |  |  |  | |  | |  |  |  |
| Heartburn |  |  |  |  |  |  | |  | |  |  |  |
|  |  |  |  |  |  |  | |  | |  |  |  |
| Constipation |  |  |  |  |  |  | |  | |  |  |  |
|  |  |  |  |  |  |  | |  | |  |  |  |
| Diarrhea |  |  |  |  |  |  | |  | |  |  |  |
|  |  |  |  |  |  |  | |  | |  |  |  |
| Dyspnea |  |  |  |  |  |  | |  | |  |  |  |
|  |  |  |  |  |  |  | |  | |  |  |  |
| Edema limbs |  |  |  |  |  |  | |  | |  |  |  |
|  |  |  |  |  |  |  | |  | |  |  |  |
| Palpitation |  |  |  |  |  |  | |  | |  |  |  |
|  |  |  |  |  |  |  | |  | |  |  |  |
| Breast or chest wall mass |  |  |  |  |  |  | |  | |  |  |  |
|  |  |  |  |  |  |  | |  | |  |  |  |
| Lymph node enlargement |  |  |  |  |  |  | |  | |  |  |  |
|  |  |  |  |  |  |  | |  | |  |  |  |
| Rash |  |  |  |  |  |  | |  | |  |  |  |
|  |  |  |  |  |  |  | |  | |  |  |  |
| Hair loss |  |  |  |  |  |  | |  | |  |  |  |
|  |  |  |  |  |  |  | |  | |  |  |  |
| Hand and-Foot syndrome |  |  |  |  |  |  | |  | |  |  |  |
|  |  |  |  |  |  |  | |  | |  |  |  |
| Paronychia |  |  |  |  |  |  | |  | |  |  |  |
|  |  |  |  |  |  |  | |  | |  |  |  |
| Injection site reaction |  |  |  |  |  |  | |  | |  |  |  |
|  |  |  |  |  |  |  | |  | |  |  |  |
| Peripheral neuropathy |  |  |  |  |  |  | |  | |  |  |  |
|  |  |  |  |  |  |  | |  | |  |  |  |
| Dizziness |  |  |  |  |  |  | |  | |  |  |  |
|  |  |  |  |  |  |  | |  | |  |  |  |
| Watery eyes |  |  |  |  |  |  | |  | |  |  |  |
|  |  |  |  |  |  |  | |  | |  |  |  |
| Pain |  |  |  |  |  |  | |  | |  |  |  |
|  |  |  |  |  |  |  | |  | |  |  |  |
| Insomnia |  |  |  |  |  |  | |  | |  |  |  |
|  |  |  |  |  |  |  | |  | |  |  |  |
| Fatigue |  |  |  |  |  |  | |  | |  |  |  |
|  |  |  |  |  |  |  | |  | |  |  |  |
| Dysuria |  |  |  |  |  |  | |  | |  |  |  |
|  |  |  |  |  |  |  | |  | |  |  |  |
| Hot flashes |  |  |  |  |  |  | |  | |  |  |  |
|  |  |  |  |  |  |  | |  | |  |  |  |
| Fever |  |  |  |  |  |  | |  | |  |  |  |
|  |  |  |  |  |  |  | |  | |  |  |  |
| Bleeding |  |  |  |  |  |  | |  | |  |  |  |
|  |  |  |  |  |  |  | |  | |  |  |  |

| 2) Urgency of intervention  (The extent to which intervention is necessary, given how quickly the problem needs to be resolved) | Not at all | | | | | |  | | Very much | | | |
| --- | --- | --- | --- | --- | --- | --- | --- | --- | --- | --- | --- | --- |
|  |  | | | | | | | | | | | |
|  | 1 | 2 | 3 | 4 | 5 | 6 | | 7 | | 8 | 9 |  |
| Oral/Pharyngeal mucositis |  |  |  |  |  |  | |  | |  |  |  |
|  |  |  |  |  |  |  | |  | |  |  |  |
| Anorexia |  |  |  |  |  |  | |  | |  |  |  |
|  |  |  |  |  |  |  | |  | |  |  |  |
| Weight loss |  |  |  |  |  |  | |  | |  |  |  |
|  |  |  |  |  |  |  | |  | |  |  |  |
| Nausea |  |  |  |  |  |  | |  | |  |  |  |
|  |  |  |  |  |  |  | |  | |  |  |  |
| Vomiting |  |  |  |  |  |  | |  | |  |  |  |
|  |  |  |  |  |  |  | |  | |  |  |  |
| Heartburn |  |  |  |  |  |  | |  | |  |  |  |
|  |  |  |  |  |  |  | |  | |  |  |  |
| Constipation |  |  |  |  |  |  | |  | |  |  |  |
|  |  |  |  |  |  |  | |  | |  |  |  |
| Diarrhea |  |  |  |  |  |  | |  | |  |  |  |
|  |  |  |  |  |  |  | |  | |  |  |  |
| Dyspnea |  |  |  |  |  |  | |  | |  |  |  |
|  |  |  |  |  |  |  | |  | |  |  |  |
| Edema limbs |  |  |  |  |  |  | |  | |  |  |  |
|  |  |  |  |  |  |  | |  | |  |  |  |
| Palpitation |  |  |  |  |  |  | |  | |  |  |  |
|  |  |  |  |  |  |  | |  | |  |  |  |
| Breast or chest wall mass |  |  |  |  |  |  | |  | |  |  |  |
|  |  |  |  |  |  |  | |  | |  |  |  |
| Lymph node enlargement |  |  |  |  |  |  | |  | |  |  |  |
|  |  |  |  |  |  |  | |  | |  |  |  |
| Rash |  |  |  |  |  |  | |  | |  |  |  |
|  |  |  |  |  |  |  | |  | |  |  |  |
| Hair loss |  |  |  |  |  |  | |  | |  |  |  |
|  |  |  |  |  |  |  | |  | |  |  |  |
| Hand and-Foot syndrome |  |  |  |  |  |  | |  | |  |  |  |
|  |  |  |  |  |  |  | |  | |  |  |  |
| Paronychia |  |  |  |  |  |  | |  | |  |  |  |
|  |  |  |  |  |  |  | |  | |  |  |  |
| Injection site reaction |  |  |  |  |  |  | |  | |  |  |  |
|  |  |  |  |  |  |  | |  | |  |  |  |
| Peripheral neuropathy |  |  |  |  |  |  | |  | |  |  |  |
|  |  |  |  |  |  |  | |  | |  |  |  |
| Dizziness |  |  |  |  |  |  | |  | |  |  |  |
|  |  |  |  |  |  |  | |  | |  |  |  |
| Watery eyes |  |  |  |  |  |  | |  | |  |  |  |
|  |  |  |  |  |  |  | |  | |  |  |  |
| Pain |  |  |  |  |  |  | |  | |  |  |  |
|  |  |  |  |  |  |  | |  | |  |  |  |
| Insomnia |  |  |  |  |  |  | |  | |  |  |  |
|  |  |  |  |  |  |  | |  | |  |  |  |
| Fatigue |  |  |  |  |  |  | |  | |  |  |  |
|  |  |  |  |  |  |  | |  | |  |  |  |
| Dysuria |  |  |  |  |  |  | |  | |  |  |  |
|  |  |  |  |  |  |  | |  | |  |  |  |
| Hot flashes |  |  |  |  |  |  | |  | |  |  |  |
|  |  |  |  |  |  |  | |  | |  |  |  |
| Fever |  |  |  |  |  |  | |  | |  |  |  |
|  |  |  |  |  |  |  | |  | |  |  |  |
| Bleeding |  |  |  |  |  |  | |  | |  |  |  |
|  |  |  |  |  |  |  | |  | |  |  |  |

| 3) Usefulness of symptom management  (Consider whether there are effective ways to manage symptoms, and how much side effects can be improved and quality of life improved by symptom management) | Not at all | | | | | |  | | Very much | | | |
| --- | --- | --- | --- | --- | --- | --- | --- | --- | --- | --- | --- | --- |
|  |  | | | | | | | | | | | |
|  | 1 | 2 | 3 | 4 | 5 | 6 | | 7 | | 8 | 9 |  |
| Oral/Pharyngeal mucositis |  |  |  |  |  |  | |  | |  |  |  |
|  |  |  |  |  |  |  | |  | |  |  |  |
| Anorexia |  |  |  |  |  |  | |  | |  |  |  |
|  |  |  |  |  |  |  | |  | |  |  |  |
| Weight loss |  |  |  |  |  |  | |  | |  |  |  |
|  |  |  |  |  |  |  | |  | |  |  |  |
| Nausea |  |  |  |  |  |  | |  | |  |  |  |
|  |  |  |  |  |  |  | |  | |  |  |  |
| Vomiting |  |  |  |  |  |  | |  | |  |  |  |
|  |  |  |  |  |  |  | |  | |  |  |  |
| Heartburn |  |  |  |  |  |  | |  | |  |  |  |
|  |  |  |  |  |  |  | |  | |  |  |  |
| Constipation |  |  |  |  |  |  | |  | |  |  |  |
|  |  |  |  |  |  |  | |  | |  |  |  |
| Diarrhea |  |  |  |  |  |  | |  | |  |  |  |
|  |  |  |  |  |  |  | |  | |  |  |  |
| Dyspnea |  |  |  |  |  |  | |  | |  |  |  |
|  |  |  |  |  |  |  | |  | |  |  |  |
| Edema limbs |  |  |  |  |  |  | |  | |  |  |  |
|  |  |  |  |  |  |  | |  | |  |  |  |
| Palpitation |  |  |  |  |  |  | |  | |  |  |  |
|  |  |  |  |  |  |  | |  | |  |  |  |
| Breast or chest wall mass |  |  |  |  |  |  | |  | |  |  |  |
|  |  |  |  |  |  |  | |  | |  |  |  |
| Lymph node enlargement |  |  |  |  |  |  | |  | |  |  |  |
|  |  |  |  |  |  |  | |  | |  |  |  |
| Rash |  |  |  |  |  |  | |  | |  |  |  |
|  |  |  |  |  |  |  | |  | |  |  |  |
| Hair loss |  |  |  |  |  |  | |  | |  |  |  |
|  |  |  |  |  |  |  | |  | |  |  |  |
| Hand and-Foot syndrome |  |  |  |  |  |  | |  | |  |  |  |
|  |  |  |  |  |  |  | |  | |  |  |  |
| Paronychia |  |  |  |  |  |  | |  | |  |  |  |
|  |  |  |  |  |  |  | |  | |  |  |  |
| Injection site reaction |  |  |  |  |  |  | |  | |  |  |  |
|  |  |  |  |  |  |  | |  | |  |  |  |
| Peripheral neuropathy |  |  |  |  |  |  | |  | |  |  |  |
|  |  |  |  |  |  |  | |  | |  |  |  |
| Dizziness |  |  |  |  |  |  | |  | |  |  |  |
|  |  |  |  |  |  |  | |  | |  |  |  |
| Watery eyes |  |  |  |  |  |  | |  | |  |  |  |
|  |  |  |  |  |  |  | |  | |  |  |  |
| Pain |  |  |  |  |  |  | |  | |  |  |  |
|  |  |  |  |  |  |  | |  | |  |  |  |
| Insomnia |  |  |  |  |  |  | |  | |  |  |  |
|  |  |  |  |  |  |  | |  | |  |  |  |
| Fatigue |  |  |  |  |  |  | |  | |  |  |  |
|  |  |  |  |  |  |  | |  | |  |  |  |
| Dysuria |  |  |  |  |  |  | |  | |  |  |  |
|  |  |  |  |  |  |  | |  | |  |  |  |
| Hot flashes |  |  |  |  |  |  | |  | |  |  |  |
|  |  |  |  |  |  |  | |  | |  |  |  |
| Fever |  |  |  |  |  |  | |  | |  |  |  |
|  |  |  |  |  |  |  | |  | |  |  |  |
| Bleeding |  |  |  |  |  |  | |  | |  |  |  |
|  |  |  |  |  |  |  | |  | |  |  |  |

3. Endocrine therapy

| 1) Significance of symptoms  (How great and important the effect of symptoms on the health status and life of cancer patients) | Not at all | | | | | |  | | Very much | | | |
| --- | --- | --- | --- | --- | --- | --- | --- | --- | --- | --- | --- | --- |
|  |  | | | | | | | | | | | |
|  | 1 | 2 | 3 | 4 | 5 | 6 | | 7 | | 8 | 9 |  |
| Oral/Pharyngeal mucositis |  |  |  |  |  |  | |  | |  |  |  |
|  |  |  |  |  |  |  | |  | |  |  |  |
| Anorexia |  |  |  |  |  |  | |  | |  |  |  |
|  |  |  |  |  |  |  | |  | |  |  |  |
| Weight loss |  |  |  |  |  |  | |  | |  |  |  |
|  |  |  |  |  |  |  | |  | |  |  |  |
| Nausea |  |  |  |  |  |  | |  | |  |  |  |
|  |  |  |  |  |  |  | |  | |  |  |  |
| Vomiting |  |  |  |  |  |  | |  | |  |  |  |
|  |  |  |  |  |  |  | |  | |  |  |  |
| Heartburn |  |  |  |  |  |  | |  | |  |  |  |
|  |  |  |  |  |  |  | |  | |  |  |  |
| Constipation |  |  |  |  |  |  | |  | |  |  |  |
|  |  |  |  |  |  |  | |  | |  |  |  |
| Diarrhea |  |  |  |  |  |  | |  | |  |  |  |
|  |  |  |  |  |  |  | |  | |  |  |  |
| Dyspnea |  |  |  |  |  |  | |  | |  |  |  |
|  |  |  |  |  |  |  | |  | |  |  |  |
| Edema limbs |  |  |  |  |  |  | |  | |  |  |  |
|  |  |  |  |  |  |  | |  | |  |  |  |
| Palpitation |  |  |  |  |  |  | |  | |  |  |  |
|  |  |  |  |  |  |  | |  | |  |  |  |
| Breast or chest wall mass |  |  |  |  |  |  | |  | |  |  |  |
|  |  |  |  |  |  |  | |  | |  |  |  |
| Lymph node enlargement |  |  |  |  |  |  | |  | |  |  |  |
|  |  |  |  |  |  |  | |  | |  |  |  |
| Rash |  |  |  |  |  |  | |  | |  |  |  |
|  |  |  |  |  |  |  | |  | |  |  |  |
| Hair loss |  |  |  |  |  |  | |  | |  |  |  |
|  |  |  |  |  |  |  | |  | |  |  |  |
| Hand and-Foot syndrome |  |  |  |  |  |  | |  | |  |  |  |
|  |  |  |  |  |  |  | |  | |  |  |  |
| Paronychia |  |  |  |  |  |  | |  | |  |  |  |
|  |  |  |  |  |  |  | |  | |  |  |  |
| Injection site reaction |  |  |  |  |  |  | |  | |  |  |  |
|  |  |  |  |  |  |  | |  | |  |  |  |
| Peripheral neuropathy |  |  |  |  |  |  | |  | |  |  |  |
|  |  |  |  |  |  |  | |  | |  |  |  |
| Dizziness |  |  |  |  |  |  | |  | |  |  |  |
|  |  |  |  |  |  |  | |  | |  |  |  |
| Watery eyes |  |  |  |  |  |  | |  | |  |  |  |
|  |  |  |  |  |  |  | |  | |  |  |  |
| Pain |  |  |  |  |  |  | |  | |  |  |  |
|  |  |  |  |  |  |  | |  | |  |  |  |
| Insomnia |  |  |  |  |  |  | |  | |  |  |  |
|  |  |  |  |  |  |  | |  | |  |  |  |
| Fatigue |  |  |  |  |  |  | |  | |  |  |  |
|  |  |  |  |  |  |  | |  | |  |  |  |
| Dysuria |  |  |  |  |  |  | |  | |  |  |  |
|  |  |  |  |  |  |  | |  | |  |  |  |
| Hot flashes |  |  |  |  |  |  | |  | |  |  |  |
|  |  |  |  |  |  |  | |  | |  |  |  |
| Fever |  |  |  |  |  |  | |  | |  |  |  |
|  |  |  |  |  |  |  | |  | |  |  |  |
| Bleeding |  |  |  |  |  |  | |  | |  |  |  |
|  |  |  |  |  |  |  | |  | |  |  |  |

| 2) Urgency of intervention  (The extent to which intervention is necessary, given how quickly the problem needs to be resolved) | Not at all | | | | | |  | | Very much | | | |
| --- | --- | --- | --- | --- | --- | --- | --- | --- | --- | --- | --- | --- |
|  |  | | | | | | | | | | | |
|  | 1 | 2 | 3 | 4 | 5 | 6 | | 7 | | 8 | 9 |  |
| Oral/Pharyngeal mucositis |  |  |  |  |  |  | |  | |  |  |  |
|  |  |  |  |  |  |  | |  | |  |  |  |
| Anorexia |  |  |  |  |  |  | |  | |  |  |  |
|  |  |  |  |  |  |  | |  | |  |  |  |
| Weight loss |  |  |  |  |  |  | |  | |  |  |  |
|  |  |  |  |  |  |  | |  | |  |  |  |
| Nausea |  |  |  |  |  |  | |  | |  |  |  |
|  |  |  |  |  |  |  | |  | |  |  |  |
| Vomiting |  |  |  |  |  |  | |  | |  |  |  |
|  |  |  |  |  |  |  | |  | |  |  |  |
| Heartburn |  |  |  |  |  |  | |  | |  |  |  |
|  |  |  |  |  |  |  | |  | |  |  |  |
| Constipation |  |  |  |  |  |  | |  | |  |  |  |
|  |  |  |  |  |  |  | |  | |  |  |  |
| Diarrhea |  |  |  |  |  |  | |  | |  |  |  |
|  |  |  |  |  |  |  | |  | |  |  |  |
| Dyspnea |  |  |  |  |  |  | |  | |  |  |  |
|  |  |  |  |  |  |  | |  | |  |  |  |
| Edema limbs |  |  |  |  |  |  | |  | |  |  |  |
|  |  |  |  |  |  |  | |  | |  |  |  |
| Palpitation |  |  |  |  |  |  | |  | |  |  |  |
|  |  |  |  |  |  |  | |  | |  |  |  |
| Breast or chest wall mass |  |  |  |  |  |  | |  | |  |  |  |
|  |  |  |  |  |  |  | |  | |  |  |  |
| Lymph node enlargement |  |  |  |  |  |  | |  | |  |  |  |
|  |  |  |  |  |  |  | |  | |  |  |  |
| Rash |  |  |  |  |  |  | |  | |  |  |  |
|  |  |  |  |  |  |  | |  | |  |  |  |
| Hair loss |  |  |  |  |  |  | |  | |  |  |  |
|  |  |  |  |  |  |  | |  | |  |  |  |
| Hand and-Foot syndrome |  |  |  |  |  |  | |  | |  |  |  |
|  |  |  |  |  |  |  | |  | |  |  |  |
| Paronychia |  |  |  |  |  |  | |  | |  |  |  |
|  |  |  |  |  |  |  | |  | |  |  |  |
| Injection site reaction |  |  |  |  |  |  | |  | |  |  |  |
|  |  |  |  |  |  |  | |  | |  |  |  |
| Peripheral neuropathy |  |  |  |  |  |  | |  | |  |  |  |
|  |  |  |  |  |  |  | |  | |  |  |  |
| Dizziness |  |  |  |  |  |  | |  | |  |  |  |
|  |  |  |  |  |  |  | |  | |  |  |  |
| Watery eyes |  |  |  |  |  |  | |  | |  |  |  |
|  |  |  |  |  |  |  | |  | |  |  |  |
| Pain |  |  |  |  |  |  | |  | |  |  |  |
|  |  |  |  |  |  |  | |  | |  |  |  |
| Insomnia |  |  |  |  |  |  | |  | |  |  |  |
|  |  |  |  |  |  |  | |  | |  |  |  |
| Fatigue |  |  |  |  |  |  | |  | |  |  |  |
|  |  |  |  |  |  |  | |  | |  |  |  |
| Dysuria |  |  |  |  |  |  | |  | |  |  |  |
|  |  |  |  |  |  |  | |  | |  |  |  |
| Hot flashes |  |  |  |  |  |  | |  | |  |  |  |
|  |  |  |  |  |  |  | |  | |  |  |  |
| Fever |  |  |  |  |  |  | |  | |  |  |  |
|  |  |  |  |  |  |  | |  | |  |  |  |
| Bleeding |  |  |  |  |  |  | |  | |  |  |  |
|  |  |  |  |  |  |  | |  | |  |  |  |

| 3) Usefulness of symptom management  (Consider whether there are effective ways to manage symptoms, and how much side effects can be improved and quality of life improved by symptom management) | Not at all | | | | | |  | | Very much | | | |
| --- | --- | --- | --- | --- | --- | --- | --- | --- | --- | --- | --- | --- |
|  |  | | | | | | | | | | | |
|  | 1 | 2 | 3 | 4 | 5 | 6 | | 7 | | 8 | 9 |  |
| Oral/Pharyngeal mucositis |  |  |  |  |  |  | |  | |  |  |  |
|  |  |  |  |  |  |  | |  | |  |  |  |
| Anorexia |  |  |  |  |  |  | |  | |  |  |  |
|  |  |  |  |  |  |  | |  | |  |  |  |
| Weight loss |  |  |  |  |  |  | |  | |  |  |  |
|  |  |  |  |  |  |  | |  | |  |  |  |
| Nausea |  |  |  |  |  |  | |  | |  |  |  |
|  |  |  |  |  |  |  | |  | |  |  |  |
| Vomiting |  |  |  |  |  |  | |  | |  |  |  |
|  |  |  |  |  |  |  | |  | |  |  |  |
| Heartburn |  |  |  |  |  |  | |  | |  |  |  |
|  |  |  |  |  |  |  | |  | |  |  |  |
| Constipation |  |  |  |  |  |  | |  | |  |  |  |
|  |  |  |  |  |  |  | |  | |  |  |  |
| Diarrhea |  |  |  |  |  |  | |  | |  |  |  |
|  |  |  |  |  |  |  | |  | |  |  |  |
| Dyspnea |  |  |  |  |  |  | |  | |  |  |  |
|  |  |  |  |  |  |  | |  | |  |  |  |
| Edema limbs |  |  |  |  |  |  | |  | |  |  |  |
|  |  |  |  |  |  |  | |  | |  |  |  |
| Palpitation |  |  |  |  |  |  | |  | |  |  |  |
|  |  |  |  |  |  |  | |  | |  |  |  |
| Breast or chest wall mass |  |  |  |  |  |  | |  | |  |  |  |
|  |  |  |  |  |  |  | |  | |  |  |  |
| Lymph node enlargement |  |  |  |  |  |  | |  | |  |  |  |
|  |  |  |  |  |  |  | |  | |  |  |  |
| Rash |  |  |  |  |  |  | |  | |  |  |  |
|  |  |  |  |  |  |  | |  | |  |  |  |
| Hair loss |  |  |  |  |  |  | |  | |  |  |  |
|  |  |  |  |  |  |  | |  | |  |  |  |
| Hand and-Foot syndrome |  |  |  |  |  |  | |  | |  |  |  |
|  |  |  |  |  |  |  | |  | |  |  |  |
| Paronychia |  |  |  |  |  |  | |  | |  |  |  |
|  |  |  |  |  |  |  | |  | |  |  |  |
| Injection site reaction |  |  |  |  |  |  | |  | |  |  |  |
|  |  |  |  |  |  |  | |  | |  |  |  |
| Peripheral neuropathy |  |  |  |  |  |  | |  | |  |  |  |
|  |  |  |  |  |  |  | |  | |  |  |  |
| Dizziness |  |  |  |  |  |  | |  | |  |  |  |
|  |  |  |  |  |  |  | |  | |  |  |  |
| Watery eyes |  |  |  |  |  |  | |  | |  |  |  |
|  |  |  |  |  |  |  | |  | |  |  |  |
| Pain |  |  |  |  |  |  | |  | |  |  |  |
|  |  |  |  |  |  |  | |  | |  |  |  |
| Insomnia |  |  |  |  |  |  | |  | |  |  |  |
|  |  |  |  |  |  |  | |  | |  |  |  |
| Fatigue |  |  |  |  |  |  | |  | |  |  |  |
|  |  |  |  |  |  |  | |  | |  |  |  |
| Dysuria |  |  |  |  |  |  | |  | |  |  |  |
|  |  |  |  |  |  |  | |  | |  |  |  |
| Hot flashes |  |  |  |  |  |  | |  | |  |  |  |
|  |  |  |  |  |  |  | |  | |  |  |  |
| Fever |  |  |  |  |  |  | |  | |  |  |  |
|  |  |  |  |  |  |  | |  | |  |  |  |
| Bleeding |  |  |  |  |  |  | |  | |  |  |  |

4. If the symptoms and side effects of a breast cancer patient are Grade ≥3 (CTCAE version 5.0), which is difficult for the patient to manage on their own, and you think that this is a case that requires treatment in the emergency room, please select ‘Agree’ If you think you do not need treatment in the emergency room, please mark one of the required management methods among ‘do not agree’.

| Toxicity | Criteria for emergency room visits (Grade ≥ 3) | Agree | Do not agree | | |
| --- | --- | --- | --- | --- | --- |
|  |  |  | Outpatient visit | Nurse counseling | Self-management |
| Anorexia | Severe weight loss or malnutrition due to insufficient calorie or fluid intake (requiring tube feeding or intravenous feeding) |  |  |  |  |
| Weight loss | Weight loss of more than 20% (requires tube feeding or intravenous nutrition) |  |  |  |  |
| Nausea | Inability to consume calories or fluids (requires tube feeding or intravenous feeding; requires hospitalization) |  |  |  |  |
| Vomiting | Vomiting 6 or more times per day (requires tube feeding or intravenous feeding; requires hospitalization) |  |  |  |  |
| Heartburn | Eating and digestive function are severely reduced (intravenous feeding or hospitalization required) |  |  |  |  |
| Constipation | Severe constipation requiring manual evacuation of stool; Difficulty doing daily life by oneself |  |  |  |  |
| Diarrhea | Defecating more than 7 times a day; Severely increased fecal output into the stoma than before; Difficulty performing daily activities on their own (requires hospitalization) |  |  |  |  |
| Dyspnea | Shortness of breath even at rest; Difficulty doing daily life by oneself |  |  |  |  |
| Cough | Severe symptoms; Difficulty doing daily life by oneself |  |  |  |  |
| Edema limbs | 30% or more difference in volume compared to the opposite side; severely out of normal anatomical contour; Difficulty doing daily life by oneself |  |  |  |  |
| Rash | Moderate or severe macules or papules on 30% or more of the body surface; Difficulty doing daily life by oneself |  |  |  |  |
| Pruritus (Itching) | Difficulty performing daily activities or sleeping on one's own due to widespread and persistent itching (requires steroids or immunosuppressants) |  |  |  |  |
| Hand and-Foot syndrome | painful severe skin changes (e.g. peeling, cracking, blistering, hemorrhage, swelling, keratosis); Difficulty doing daily life by oneself |  |  |  |  |
| Paronychia | Difficulty performing daily activities on their own (requires surgical intervention; requires injectable antibiotics) |  |  |  |  |
| Injection site reaction | Ulceration or necrosis, with severe tissue damage (requires surgical intervention) |  |  |  |  |
| Peripheral sensory neuropathy | Severe symptoms; Difficulty doing daily life by oneself |  |  |  |  |
| Dizziness | Feeling extremely unstable or moving; Difficulty doing daily life by oneself |  |  |  |  |
| Watering eyes | Visual acuity is markedly reduced |  |  |  |  |
| Blurred vision | Visual acuity is significantly reduced; Difficulty doing daily life by oneself |  |  |  |  |
| Pain | Severe pain; Difficulty doing daily life by oneself |  |  |  |  |
| Insomnia | Difficulty falling asleep, waking up frequently, or waking up early |  |  |  |  |
| Fatigue | Fatigue even after rest, difficulty performing daily activities on their own |  |  |  |  |
| Anxiety | Severe symptoms; Difficulty performing daily activities on their own; hospitalization required |  |  |  |  |
| Depression | Severe depressive symptoms; Difficulty doing daily life by oneself |  |  |  |  |
| Dyspareunia | Discomfort or pain during vaginal passage (discomfort or pain that is not relieved by vaginal lubricants or estrogen) |  |  |  |  |
| Hot flashes | Severe symptoms; Difficulty doing daily life by oneself |  |  |  |  |
| Fever | Fever above 38 degrees lasting less than 24 hours |  |  |  |  |
| Bleeding | Uncontrolled bleeding (requires blood transfusion; requires invasive procedures) |  |  |  |  |

4. Please comment if there are additional symptoms that should be included to manage symptoms and side effects of breast cancer patients.

|  |
| --- |

5. Please comment if there are any aspects to be considered in a web-based program for managing symptoms and side effects of breast cancer patients.

|  |
| --- |

**You have completed Round 1 of the Delphi Survey.**

**Thank you very much for participating in the survey.**
