# Supplementary material for: Development and pilot study of “Smart Cancer Care”: a platform for managing side effects of chemotherapy
Source: BMC Health Serv Res. 2023 Aug 29;23:922. doi: 10.1186/s12913-023-09871-0 (PMC10466749; doi:10.1186/s12913-023-09871-0)
Supplement: Supplementary file 4 — Supplementary Material 4 [file 12913_2023_9871_MOESM4_ESM.docx]

**Supplementary file 2: Survey to determine the feasibility and usefulness of Smart Cancer Care (for patients)**

1. General characteristics of respondents

| Sex | 1) Man 2) Woman | Age | ( ) |
| --- | --- | --- | --- |
| Education level | 1) Uneducated 2) Elementary school graduate 3) Middle school graduate  4) High school graduate 5) College graduate or higher  ※ Uneducated: 1) Korean decipherable 2) Unable to decipher Korean | | |
| Marital Status | 1) Married 2) Single 3) Widowed 4) Divorced 5) Others | | |
| Occupation | 1) None 2) Yes: ( ) | | |
| Level of care | 1) None 2) Spouse support 3) Family support (parents or siblings)  4) Help from social worker 5) Others: ( ) | | |
| Religion | 1) None 2) Christianity 3) Catholicism 4) Buddhism 5) Others: ( ) | | |
| Income level | Standard of household monthly income  1) ≤ 1.5 million won 2) 1.51-3 million won 3) 3.01-5 million won 4) 5 million won > | | |
| Current health status | 1) Very good 2) Good 3) Fair 4) Poor 5) Very poor | | |
| Cancer treatment side effects symptom management | 1) Know well 2) Know to some extent 3) Do not know 4) Do not know at all | | |
| Diagnosis | ( ) | | |
| Cancer stage | ( ) | | |

2. Please mark with √ in the appropriate box what you felt while using Smart Cancer Care.

|  | Item | Strongly disagree | Disagree | Neither agree nor disagree | Agree | Strongly agree |
| --- | --- | --- | --- | --- | --- | --- |
| Cognitive | I was able to understand the purpose of the program appropriately. |  |  |  |  |  |
|  | Overall, it was difficult to understand the contents of the program (reverse question). |  |  |  |  |  |
|  | The program was generally easy to use. |  |  |  |  |  |
| Psychological | I feel uncomfortable entering my treatment status into the program (reverse question). |  |  |  |  |  |
|  | I would feel comfortable if I managed side effects using the program |  |  |  |  |  |
|  | I would recommend using this program for cancer patients around me. |  |  |  |  |  |
| Written | The contents entered in the program reflect my treatment status properly. |  |  |  |  |  |
|  | There are too many items to respond to in the program's questionnaire. |  |  |  |  |  |
|  | I need help from someone else to use the program. |  |  |  |  |  |
| Social | The use of a management program for side effect symptoms will reduce hospital visits. |  |  |  |  |  |
|  | The program will be helpful when seeing medical staff. |  |  |  |  |  |
|  | I hope the program will be widely used. |  |  |  |  |  |

3. Experience using Smart Cancer Care (open-ended questions, recording of answers)

[Key question] How was it when you used the app?

→ Was it helpful in managing side effects symptoms by comparing before and after using the app?

→ If so, what was the most helpful?

→ What was the most uncomfortable part of using the app?

→ How would you like to correct the inconvenient part?

→ Did you understand the contents of the app?

→ How understandable is the anti-cancer side effect self-management program using the app?

→ Was it difficult to use even by yourself?

→ What was difficult about using it alone?

→ How was the researcher helpful?

[Probing question]

→ Were there any difficulties with downloading?

→ Were there any difficulties in implementation?

→ Did you have any difficulties logging in?

→ Were there any problems with entering your cancer diagnosis and anticancer drug information?

→ Were there any problems with side effect symptom selection?

→ Were there any problems with entering symptoms of side effects?

→ Were the side effect symptom assessment results expected?

→ Was the self-management content easy to apply?

→ Are there any details or features you would like to see added to the app?

→ Are you willing to continue using the app in the future?

→ Would you recommend using the app to others?

**The survey is completed.**

**Thank you for taking the time to complete this survey.**

**Survey to determine the feasibility and usefulness of Smart Cancer Care (for medical staff)**

1. General characteristics of respondents

| Sex | 1) Man 2) Woman |
| --- | --- |
| Occupation | 1) General practitioner 2) Resident 3) Specialist 4) Nurse |
| Cancer treatment side effects symptom management | 1) Know well 2) Know to some extent 3) Do not know 4) Do not know at all |
| Experience with cancer-related apps | 1) Yes 2) No |

2. Please mark with √ in the appropriate box what you felt while using Smart Cancer Care.

|  | Item | Strongly disagree | Disagree | Neither agree nor disagree | Agree | Strongly agree |
| --- | --- | --- | --- | --- | --- | --- |
| Cognitive | I was able to understand the purpose of the program appropriately. |  |  |  |  |  |
|  | Overall, it was difficult to understand the contents of the program (reverse question). |  |  |  |  |  |
|  | The program was generally easy to use. |  |  |  |  |  |
| Psychological | I feel uncomfortable to check patient-related information through the program (reverse question). |  |  |  |  |  |
|  | I would feel comfortable if the patients use the program to manage their side effect symptoms |  |  |  |  |  |
|  | I would recommend using this program for the medical staff around me. |  |  |  |  |  |
| Written | Patient information available through the program is appropriate. |  |  |  |  |  |
|  | By checking the symptoms of the patient through the program, it helps to take appropriate measures for the patients. |  |  |  |  |  |
|  | Additional functions are required to use the program. |  |  |  |  |  |
| Social | The program will reduce the overall burden of managing side effect symptoms on patients. |  |  |  |  |  |
|  | The program will be helpful to care for patients. |  |  |  |  |  |
|  | I hope the program will be widely used. |  |  |  |  |  |

3. Experience using Smart Cancer Care (open-ended questions, recording of answers)

[Key question] How was it when you used the app? How was it to check the patient's condition using the program?

→ What was the most uncomfortable part of using the program?

→ How would you like to correct the inconvenient part?

→ Has the comparison of before and after using the program helped the patient manage side effects?

→ What, if any, was most helpful?

→ Did you understand the contents of the program?

→ How understandable is the anti-cancer side effect self-management program using the program?”

→ Was there anything else you would like to see added to the program?”

→ Are you willing to continue using the program in the future?”

→ Would you recommend others to use the program?”

**The survey is completed.**

**Thank you for taking the time to complete this survey.**
