# Supplementary material for: Development and pilot study of “Smart Cancer Care”: a platform for managing side effects of chemotherapy
Source: BMC Health Serv Res. 2023 Aug 29;23:922. doi: 10.1186/s12913-023-09871-0 (PMC10466749; doi:10.1186/s12913-023-09871-0)
Supplement: Supplementary file 5 — Supplementary Material 5 [file 12913_2023_9871_MOESM5_ESM.docx]

**Supplementary file 3: Major side effects due to chemotherapy and corresponding management guidelines**

**Ⅰ. Oral symptoms**

**1. Stomatitis**

**Grade-1 (level 1): mild stage**

• Observe changes in oral condition frequently and gargle 3 to 4 times a day after brushing. Do this for about 30 seconds at a time and avoid eating within 30 minutes. For gargling solution, use sodium bicarbonate gargle or physiological saline solution.

• Sodium bicarbonate gargle: 1/2 teaspoon of salt + 1 teaspoon of soda + 250cc of water, be sure to rinse your mouth with water after gargling.

• Physiological saline solution: You can mix 1/4 teaspoon of salt in 500 ml of water.

• Chlorhexidine-based disinfectant gargles are not used prophylactically.

• Do not use commercially available mouthwashes containing alcohol.

• Gargling solution should be at room temperature, not too cold or too hot.

Use a soft-bristled toothbrush or soak in warm water.

• Use fluoride toothpaste to prevent cavities and replace brush heads regularly.

• If you are unable to brush your teeth by yourself, wipe your mouth with cotton or gauze soaked in physiological saline, or use a syringe or other tool to rinse your mouth with your head sideways.

• See your dentist for problems that could cause irritation in your mouth.

• Floss daily unless thrombocytopenia is present.

• Avoid hot, spicy, harsh and sour foods.

• Dentures should only be used for eating when possible, soaked in a foaming cleanser and rinsed before use.

• Apply a generous amount of moisturizer around the lips.

• If dry mouth is accompanied by frequent hydration, chew xylitol gum or use artificial saliva before bed.

• Supplement with vitamin C if oral intake is not adequate.

**Grade-2 (2nd level): moderate stage**

• Observe changes in the condition of the mouth frequently by shining a light on it, and gargle (sodium bicarbonate gargle or physiological saline gargle) every 2 hours.

• Do not use disinfectant gargling solution or pain relief gargling solution more than 2-3 times a day.

• Food is blended or gently cooked and eaten in small amounts and often.

• Dry mouth can make stomatitis worse, so drink plenty of fluids and use artificial saliva.

• If it is difficult to eat properly due to pain in the mouth, take additional nutritional beverages.

• Applying ice before eating or using a local anesthetic gargling solution (not rinsing with water after gargling) can help relieve pain. After gargling with a local anesthetic, the mouth may become numb and burn, so hot food should be avoided and care should be taken not to aspirate water.

• Take your temperature frequently and go to the emergency room if you have a persistent high fever (38 degrees or higher).

• If there is bleeding, rinse your mouth with cold water or apply pressure with gauze soaked in ice water. (If the platelet count is low, hemostasis may not work well, so if bleeding continues, do a blood test.)

• If symptoms become severe, treatment with antibiotics (or antifungals) or anti-inflammatory or narcotic drugs to prevent sepsis and dehydration and intravenous nutrition may be required.

**Grade-3 (level 3): severe stage; emergency room visit or outpatient visit or nurse consultation**

• Severe pain making it difficult to eat or drink; Difficulty doing daily life by oneself

• Stomach cancer: emergency room

• Colorectal cancer: outpatient

• Lung cancer: outpatient

• Breast Cancer: outpatient

**2. Dry mouth, xerostomia**

**Grade-1 (level 1): mild stage**

• Frequently observe changes in the oral condition and gargle after brushing (normal saline solution or water mixed with 1/4 teaspoon of salt in 500ml) 3 to 4 times a day. Do this for about 30 seconds at a time and avoid eating within 30 minutes. The mouthwash should be at room temperature, not too cold or too hot.

• Avoid mouthwashes that contain alcohol, as they can aggravate dry mouth.

• Use a soft-bristled toothbrush or soak in warm water.

• If you cannot brush your teeth by yourself, wipe your mouth frequently with cotton or gauze soaked in physiological saline.

• See your dentist for problems that could cause irritation in your mouth.

• Brush with fluoride-containing toothpaste and floss daily unless thrombocytopenia is present.

• Avoid hot, spicy, harsh and sour foods.

• Dentures should only be used for eating when possible, soaked in a foaming cleanser and rinsed before use.

• Apply a generous amount of moisturizer around the lips.

• Increase fluid intake (8 to 10 glasses of water per day) and supplement with vitamin C if oral intake is not adequate.

• Be careful not to get caught when drinking water. (It is helpful to hold a glass of water and swallow with your head tilted slightly forward.)

• Avoid dry environments such as excessive heating in winter and use a humidifier.

**Grade-2 (2nd level): moderate stage**

• Drink fluids frequently between meals and use artificial saliva (which forms a slippery barrier in the mouth) before going to bed.

• Chewing xylitol gum can stimulate masticatory movements and salivation.

• Use a spray to spray water into the mouth from time to time.

• Avoid dry food and try eating with meat broth or sauce.

• Eat cold soda, such as lemonade, soda water, orange juice, pineapple, or unsweetened candy.

• Your dentures may not fit well, so get them taken care of by your dentist.

• Take additional nutritional drinks for protein supplementation.

• In case of severe symptoms, medical treatment may be required, so consult with your doctor.

**Grade-3 (level 3): Not applicable.**

**Ⅱ. Digestive symptoms**

**1. Loss of appetite**

**Grade-1 (level 1): mild stage**

• In the past week, if there is no loss of appetite and there are no restrictions in daily life, proceed with preventive management.

• Tell your family what foods you like to eat.

• If you don't have an appetite, you can substitute food such as milkshakes or juices.

• Do not eat alone, enjoy a meal with family or friends.

• If you don't like the smell of cooking food, avoid the place.

• Try light exercise before meals.

• Try new foods.

• Sour taste stimulates the taste buds, so sour fruit juices such as lemonade or orange juice are helpful if you do not have mouth sores.

**Grade-2 (2nd level): moderate stage**

• You have had a slight loss of appetite or limitations in your daily life in the past week.

• Divide it into 5-6 meals a day and eat it in small plates.

• Avoid drinking water 30 minutes before meals. Avoid cabbage or carbonated drinks (cider, cola) as much as possible, as they cause abdominal distension due to gas.

• During mealtime, do not rush and chew slowly.

• Prepare foods you like or want to eat in advance, divide them into small portions, and store them in the freezer.

• Keep food within easy reach and eat whenever you feel like it.

• Plan your meals for the day ahead of time. I usually have the best appetite in the morning, so make sure to have breakfast. Consider eating 1/3 of your daily protein and calorie intake in the morning.

• Regularly take care of your mouth and rinse your mouth thoroughly before and after meals.

• Avoid mixing hot and cold foods.

• If you increase your usual activity and have no appetite due to pain, try taking a pain reliever before eating.

• If possible, eat away from the smelly kitchen.

• Get into the habit of eating regularly and on time.

• It is recommended to use seasonings that reduce the smell of the ingredients and stimulate the appetite.

• If the amount of food is insufficient, use nutritional supplements in the form of drinking (New Care, Harmonylan, Greenvia, Encover, etc.)

• Try to eat foods that are easy to eat and high in calories: for example, increase your intake of dairy products such as puddings, jellies, ice cream, yogurt, and milkshakes, as well as poached eggs, cheese, milk, eggs, beans, meat, and nuts. do.

**Grade-3 (level 3): severe stage; emergency room visit or outpatient visit or nurse consultation**

• Severe weight loss or malnutrition due to lack of food or water intake.

• Gastric cancer: outpatient

• Colorectal cancer: outpatient

• Lung cancer: outpatient

• Breast Cancer: outpatient

**2. Nausea, Vomiting**

**Grade-1 (level 1): mild stage**

• In the past week, no nausea or vomiting: no restrictions in daily activities. Preventative management is required.

• Open windows to ventilate and get some fresh air. If you feel nauseous, relax and breathe slowly and deeply.

• Do not lie down immediately after eating. If you need to take a break, keep your upper body upright or reclined for at least 30 minutes to 1 hour.

• Wear loose-fitting clothing rather than tight clothing, and move slowly so as not to feel dizzy.

• Rinse your mouth frequently to avoid irritating the stomach to keep it fresh.

• If you have dentures, remove them before treatment.

• Placing a cool towel over your head or neck can help.

• During chemotherapy, chewing gum or candy that eliminates metallic or bitter tastes can help reduce nausea.

• It's also a good idea to focus your attention elsewhere, such as music, games, TV, meditation, or yoga, so you don't just focus on the nausea.

• Food odors from cooking may make nausea and vomiting worse.

• Constipation may cause nausea, so it is recommended to control in advance.

• It is also good to sleep when you have nausea.

**Grade-2 (2nd level): moderate stage**

• Over the past week, nausea and vomiting have been infrequent and limited in daily activities.

• Eat bland, soft, easily digestible foods.

• It is recommended to consume small amounts slowly and often.

• Taking deep breaths or holding an ice cube in your mouth will calm you down.

• Drinking water can make you feel full, so avoid too many soups or drinks with your meals.

• Hot food can make you feel nauseous, so keep drinks and food cold, and freeze your favorite beverage.

• Let the patient choose what and when they want to eat, and not force food.

• Moving right after a meal slows down digestion, so take a break after eating, and it's best to sit upright and rest for an hour after eating.

• The eating area should be well ventilated and free of unpleasant food odors.

• When you feel nauseous, you stop eating your favorite foods, and you may end up hating them forever.

• If you feel nauseous in the morning, eat toast or crackers before you wake up.

• Know when you feel nauseous and what causes it (specific food, event, background).

• Avoid greasy, fried, salty, spicy and overly sweet foods.

• Cleans the mouth and oral cavity. Brush your teeth at least twice a day, even if you are not eating. (Avoid any commercially available mouthwash that contains alcohol, as it can dry out your mouth.) When you feel a bad taste in your mouth, suck on mint candy or lozenges.

• When you can only drink water, drink honey water, sugar water, and ion drinks.

• Nausea and vomiting due to the smell of food may become severe. When this happens, try not to prepare food or smell food. Try eating at a restaurant.

• A light walk in the fresh air is also good.

• Reading, listening to your favorite music, or watching television are also good options.

• Close your eyes, breathe slowly and deeply, and think of a time when you felt good.

• Live with a happy heart, a happy heart.

• In general, for nausea and vomiting, foods such as biscuits, toast, yogurt, raw skin-on chicken, soft, bland fruits and vegetables such as canned peaches, and ice cubes are good choices.

• On the other hand, it is recommended to avoid fatty, fried foods, very sweet foods such as candy and cakes, spicy or hot foods, strong-smelling dairy products, red meat, and coffee.

**Grade-3 (level 3): severe stage; emergency room visit or outpatient visit or nurse consultation**

Nausea: inability to eat or drink

• Gastric cancer: outpatient

• Colorectal cancer: outpatient

• Lung cancer: outpatient

• Breast Cancer: outpatient

Vomiting: vomiting more than 6 times a day

• Stomach cancer: emergency room

• Colorectal cancer: emergency room

• Lung Cancer: emergency room

• Breast Cancer: emergency room

**3. Hiccups**

**Grade-1 (level 1): mild stage**

- Mild symptoms with a score of 3 or less on the numerical evaluation scale do not require medical intervention, and are managed with the goal of preventing future symptoms.

• Check if dexamethasone is administered and replace with equivalent dose of methylprednisolone.

• Avoid sudden temperature changes.

• Avoid drinking alcohol or carbonated beverages

• Avoid eating too quickly

**Grade-2 (2nd level): moderate stage**

- This is a stage where daily life performance may be limited due to moderate symptoms with a numerical evaluation score of 4 or higher, and medical intervention may be required. The goal is to control the current symptoms.

• Check if dexamethasone is administered and replace with equivalent dose of methylprednisolone.

• Breathe slowly and deeply into a paper bag.

• Hold your breath.

• Try drinking ice water slowly.

• Swallow a spoonful of sugar.

• Chew a slice of lemon.

• Give drugs such as chloropromazine, baclofen or metoclopramide if symptoms persist.

Grade-3 (level 3): severe stage

- With a numerical evaluation score of 7 or higher, it is difficult to perform daily life due to sleep disturbance or breathing difficulties.

• Check if dexamethasone is administered and replace with equivalent dose of methylprednisolone.

• Administer drugs such as chloropromazine, baclofen, or metoclopramide, and if there is no response, try gabapentin.

• Consider a phrenic nerve block if there is no response to medication.

**4. Constipation**

**Grade-1 (level 1): mild stage**

- Occasionally, there are intermittent symptoms of constipation, and preventive management is required with occasional use of stool softeners, laxatives, dietary changes, and enemas.

• Try to defecate regularly after eating and, if possible, after breakfast.

• Do not hold back when you want to have a bowel movement.

• Create a comfortable and private environment for defecation.

• Eat foods high in fiber (vegetables, greens, brown rice, nuts, etc.).

• Drink at least 8-10 glasses of fluid per day unless contraindicated.

• Keep daily activity or exercise as normal as possible (walking, biking, etc.).

• It is also good to take stool softeners prescribed by medical staff.

• If there are no contraindications, take a stool softener at the same time as taking pain relievers.

**Grade-2 (2nd level): moderate stage**

- Even if you use laxatives regularly, the symptoms of constipation persist and your daily life is restricted.

• If constipation persists for more than 2 days after lifestyle improvement, dietary fiber supplementation, or stool softener, consider adding stool softeners or using an enema or suppository after consulting with a medical professional.

• Avoid suppositories or enemas when neutrophils are significantly reduced after chemotherapy (usually between 1 and 2 weeks after chemotherapy).

**Grade-3 (level 3): severe stage; emergency room visit or outpatient visit or nurse consultation**

• Severe constipation that requires passing stool with your fingers.

• Gastric cancer: outpatient

• Colorectal cancer: outpatient

• Lung cancer: outpatient

• Breast Cancer: outpatient

**5. Soft stools (diarrhea)**

**Grade-1 (level 1): mild stage**

- The frequency of defecation is less than 4 times/day, and the amount of feces coming out of the stoma is slightly higher than usual, so preventive management is required.

• Drink plenty of fluids.

• Eat small, frequent meals because your intestines are weak.

• Foods to avoid: Foods that irritate the intestines or cause gas.

Eat beans, raw vegetables, raw fruits, corn, cabbage, soft drinks, strong spices or caffeinated beverages, sour or spicy foods, fatty or coarse-fiber vegetables, starchy foods, milk and dairy products, and foods at room temperature.

• If the skin around the anus is irritated and swollen due to diarrhea, wash the area around the anus with warm water after defecation and dry thoroughly.

• If diarrhea causes open wounds to the anus, apply a soothing cream or ointment to the wounds as they can become infected and cause pain and inflammation.

• Sitz baths are helpful if you have hemorrhoids.

**Grade-2 (2nd level): moderate stage**

- If the number of bowel movements is 4-6 times a day: The amount of stoma increases slightly than usual, and daily life is limited.

• Take anti-diarrheal medications as prescribed by your doctor.

• If you are taking oral anticancer drugs, discuss with your medical staff whether to discontinue the anticancer drugs you are taking.

• If there is no diarrhea for more than 12 hours after taking the antidiarrheal medication, stop taking the antidiarrheal medication and gradually proceed with solid food.

**Grade-3 (level 3): severe stage; emergency room visit or outpatient visit or nurse consultation**

• Defecating more than 7 times a day

• Stomach cancer: emergency room

• Colorectal cancer: outpatient

• Lung Cancer: emergency Room

• Breast Cancer: outpatient

**Ⅲ. Respiratory symptoms**

**1. Cough**

**Grade-1 (level 1): mild stage**

- It is not enough to take medicine, but coughing may occur.

• Position yourself on a side that is comfortable for breathing and avoids coughing.

• For a cough with phlegm, hydration, use of a humidifier, and saline spray may help.

**Grade-2 (2nd level): moderate stage**

- Due to coughing, daily life may be restricted, and help from medical staff may be required, such as drug prescription.

- medical management

• Take prescribed medications.

• If there is phlegm, perform physical therapy for sputum discharge that has been trained.

• Suction prescribed home oxygen.

**Grade-3 (level 3): severe stage; emergency room visit or outpatient visit or nurse consultation**

• Coughing that makes daily life difficult

• Gastric cancer: outpatient

• Colorectal cancer: not applicable

• Lung cancer: outpatient

• Breast Cancer: outpatient

**2. Shortness of breath (dyspnea)**

**Grade-1 (level 1): mild stage**

- Usually no shortness of breath, but shortness of breath occurs during moderate-intensity activity, but can work alone.

• It may help to direct or indirectly cool the face by opening a window or using a hand fan.

• Create a stable environment (comfortable position, comfortable mindset, breathing exercises), take prescribed medications, and inhale prescribed oxygen if needed.

• Do not wear clothes that are too tight.

• Ask them to record their breathing difficulties and what happened.

• Prevent symptom triggers, and set activity levels and priorities.

• Avoid environments that may aggravate shortness of breath. (Direct exposure to cold air, smoking, inadequate humidity)

• Have them record side effects from treatment.

• Do breathing exercises.

**Grade-2 (2nd level): moderate stage**

- Breathing difficulties occur even during low-intensity activities, which restricts daily life and may require oxygen equipment.

- Medical management

• Take prescribed medications such as bronchodilators, steroids, and pain relievers.

• Chemotherapy may be stopped.

• Aspirate the prescribed oxygen.

**Grade-3 (level 3): severe stage; emergency room visit or outpatient visit or nurse consultation**

• Shortness of breath even at rest; difficulty doing daily life by oneself

• Stomach cancer: emergency room

• Colorectal cancer: not applicable

• Lung Cancer: emergency Room

• Breast Cancer: emergency room

**Ⅳ. Circulatory symptoms**

**1. Lymphedema**

**Grade-1 (level 1): mild stage**

- There may be slight thickening or faint skin color change.

• Lymphedema, even the smallest swelling, should be reported to the health care provider.

• Conduct self-monitoring by continuously measuring limb circumference and observing changes in size/sensation/color/temperature/skin.

• Prevent damage and infection.

• Maintain meticulous skin hygiene and nail care.

• Beware of paper cuts or abrasions, pins, insect bites or pet scratches.

• Apply skin moisturizers and topical antibiotics as needed.

• Protect exposed skin during activities that may cause skin damage, including wearing sunscreen and gloves.

• Avoid medical procedures that may cause infection, such as vaccinations, acupuncture, phlebotomy, intravenous drips and venography, and consult your health care provider if necessary.

• Avoid exposure to extreme temperatures.

• Elevate areas where lymphedema may occur, unless contraindicated.

• Gentle elevation can reduce swelling in the early stages, avoid prolonged standing, hugging, or crossing your legs.

• Maintain ideal weight through diet and exercise.

• Avoid tight clothing, jewelry, etc. to prevent strain on the extremities.

• Blood pressure is not measured on the arm where the axillary resection was performed.

• Consult with medical staff when making flight and travel plans.

Grade-2 (2nd level): moderate stage

- Significant skin color change, leathery skin texture, papillae formation, and activity restrictions in daily life are present, but self-care is possible.

• Maintain preventive care.

• If swelling is found, the best treatment for lymphedema is complete decongestion, although there is still weak evidence.

• Perform manual lymphatic drainage as instructed.

• Apply a compression bandage or compression stockings.

• Exercise as instructed.

• Keep the skin firm and elastic by using a moisturizing cream to prevent cracking, and constantly monitor for signs of infection and treat any minor problems immediately.

• Low-level laser therapy may be performed. Non-invasive low-level (cold) laser treatment. (medical treatment)

• Maintain skin care/cleanliness.

• Follow a diet to maintain or lose weight.

• An intermittent air pump may be applied.

• If you need psychosocial support, ask your health care provider.

**Grade-3 (level 3): severe stage; emergency room visit or outpatient visit or nurse consultation**

• More than 30% difference in volume compared to the opposite side; severely out of normal anatomical contour; difficulty doing daily life by oneself

• Gastric cancer: outpatient

• Colorectal cancer: outpatient

• Lung cancer: outpatient

• Breast Cancer: outpatient

**2. Malignant pericardial effusion**

**Grade-1 (level 1): mild stage**

• Sleep with your head elevated when breathing is difficult, or use a pillow to elevate your upper body.

• Energy must be conserved. Activities are mostly in the morning, with time to unwind and relax.

• Your doctor may apply oxygen therapy if needed to help you breathe.

• Taking pain relievers can relieve chest pain and help you breathe easier.

**Grade-2 (2nd level): moderate stage**

• A feeling of tightness in the chest, rapidly progressing shortness of breath, or restlessness may require an emergency room visit.

**Grade-3 (level 3): not applicable.**

**3. Phlebitis (Extravasation)**

**Grade-1 (level 1): mild stage**

• Be sure to report any pain or abnormal sensations (pain, swelling, redness, discomfort, burning, or stabbing pain (numbness) at the injection site).

• Intravenous infusions are administered through a recent intravenous line inserted within the last 24 hours.

• Avoid veins in the fingers, wrists, and the inside of the elbow.

• Avoid areas with hardened blood vessels, blood clots, or scarring.

• Avoid vascular injections in limbs with impaired circulation or in the lower extremity of a previous venipuncture attempt.

• Select a cannula (needle) appropriate for the vessel size.

• The intravenous injection site is securely fixed to the skin using a transparent dressing and the insertion site can be observed.

• A central venous line must be used to infuse vesicular (effervescent) medications.

**Grade-2 (2nd level): moderate stage**

• Treatment depends on the drug leaking from blood vessels into tissues. Depending on the type of drug, cold and warm compresses can be applied. Consult your healthcare provider.

• Elevation of the arm: Elevating the arm does not reduce pain or resolve the phlebitis, but only elevates the phlebitis site and assists in the reabsorption of the phlebitis medication. do.

• An antidote may be used if needed.

**Grade-3 (level 3): not applicable.**

**4. Hypertension (increased blood pressure)**

**Grade-1 (level 1): mild stage**

- Systolic 120-139 mmHg, diastolic 80-89 mmHg

**Grade-2 (2nd level): moderate stage**

- Systolic 140-159 mmHg, diastolic 90-99 mmHg

**Grade-3 (level 3): severe stage**

• Systolic greater than 160 mmHg, diastolic greater than 100 mmHg

• In case of abnormal symptoms such as sudden memory loss, loss of consciousness, visual impairment, persistent headache, slurred speech, numbness in the limbs and tingling, immediately visit a hospital or emergency room.

• When taking high blood pressure medicine, you need to be careful because there is a risk of orthostatic hypotension, dizziness, nausea, and falling.

• Be aware of potential side effects of the medications you are taking and report any changes in your blood pressure to your healthcare provider. For example, to prevent high blood pressure when taking an anticancer drug called procarbazine, sometimes tyramine ingredients [cheese (blue cheese, cheddar cheese, mozzarella cheese, Parmesan cheese, Swiss cheese), yogurt, wine, beer, animal liver, sausage] may be required.

**5. Deep vein thrombosis**

**Grade-1 (level 1): mild stage**

• Be mindful of the points on long flights.

- Stand up and walk every 1-2 hours.

- Do not smoke right before the trip.

- Wear loose and comfortable clothing.

- Change your posture while sitting and move your legs and feet frequently.

- Drink plenty of fluids.

- Wear knee-high compression stockings.

**Grade-2 (2nd level): moderate stage**

**• Expert consultation is required in the following cases:**

- Severe swelling of the lower extremities or changes in skin color

- When fever and pain occur

**Grade-3 (level 3): severe stage**

• An emergency visit is needed if:

- Pain in the chest when breathing

- When you feel severe shortness of breath

**Ⅴ. Skin symptoms**

**1. Skin rashes, spots, pimples or pimples on the face or chest**

**Grade-1 (level 1): mild stage**

• Use sunscreen

• Keep your skin moist (alcohol-free lotion is best).

• If symptoms are severe due to side effects related to treatment, the dose of the drug may be reduced or stopped, so consult with the medical staff.

• It is helpful to wash your face with a spoonful of baking soda in a washbasin.

• Do not touch or squeeze pimples with your hands. Secondary infection complications may occur.

• It can be well controlled by simply washing your face and applying antibiotic ointment in severe cases.

• Bathe in cool or lukewarm water, use mild soap, and pat dry. (Do not rub)

• Use a water-based moisturizer on unbroken skin.

• Reduce skin trauma. (Use cotton products, avoid tight and rough clothes)

• If an allergic reaction is suspected, an antihistamine or calamine lotion may be prescribed.

• Fragrant face wash and laundry products are prohibited

• UV protection; Use sunscreen (SPF 15 or higher).

• Direct use of ice packs is prohibited.

• No chlorinated swimming pools or tub baths.

• Avoid shaving the irritated area.

• Take a sitz bath (for perianal lesions) if necessary.

• If possible, expose the skin to air (keep it open).

• Stays hydrated.

• Do not tape or bandage on irritated skin.

• Wash your hands often and be careful not to scratch or cut your skin.

**Grade-2 (2nd level): moderate stage**

• In case of severe acne and pus.

• If accompanied by generalized fever or chills.

• Discomfort that interferes with daily life.

• Exposure to people with a history of skin infection.

• If your symptoms are getting worse quickly.

• When a wound (surgery) is opened or inflammatory material is drained.

• If shingles is suspected.

**Grade-3 (level 3): severe stage; emergency room visit or outpatient visit or nurse consultation**

• Severe papules or pustules covering more than 30% of the body surface; local infection requiring antibiotics; Difficulty in daily life

• Gastric cancer: outpatient

• Colorectal cancer: outpatient

• Lung cancer: outpatient

• Breast Cancer: outpatient

**2. Hair Loss**

**Grade-1 (level 1): mild stage**

• If hair loss is less than 50% when viewed closely at close range;

• Wigs or hats are not needed to hide hair loss, although different hair styles may be needed to cover up hair loss.

**Grade-2 (2nd level): moderate stage**

• Easily noticeable to others and greater than 50% hair loss;

• The patient needs a wig, part wig, hair extensions, or hat to mask hair loss related to psychosocial effects.

**Grade-3 (level 3): not applicable.**

**3. Dry skin, itchy skin**

**Grade-1 (level 1): mild stage**

• Carefully observe that there are no red, rough cracks on the skin, white areas, or bleeding areas such as elbows and knees.

• Apply a few drops of mineral oil or baby oil to your bath.

• Apply moisturizer immediately after showering. Apply when the skin is still moist to prevent moisture loss.

• Avoid pushing too hard when showering or bathing.

• Avoid perfumes or toners that contain alcohol.

• Use an electric razor rather than a blade razor.

• Drink more than 2L of water per day.

• Avoid exposure to dry cold wind or heat.

**Grade-2 (2nd level): moderate stage**

• If you have dry skin, use a moisturizer twice a day.

• It is helpful to use a mixture of moisturizer and menthol ingredients. Stored in the refrigerator, itching may be relieved with a cool feeling.

• Take a cool or lukewarm shower as hot water can make itching worse.

• Using baking soda instead of soap helps with itching.

• Use a moisturizer after showering and before drying.

• Keep nails clean and short and rub rather than scratch.

• Avoid chemical detergents, strongly scented soaps, bubble baths, and products containing lanolin, which can cause allergies, on the itchy area.

• Wear clothes made of natural fibers such as cotton.

• Keep the room cool.

• Avoid alcohol and spicy food.

• Subcutaneous Electrical Nerve Stimulation (TENS) may be helpful.

**Grade-3 (level 3): severe stage; emergency room visit or outpatient visit or nurse consultation**

• Difficulty performing daily activities or sleeping due to widespread and persistent itching (needs steroids or immunesuppressants)

• Gastric cancer: outpatient

• Colorectal cancer: nurse consultation

• Lung cancer: outpatient

• Breast cancer: outpatient

**4. Rashes on the hands or feet, cracking, peeling, red, and painful (hand-foot syndrome)**

**Grade-1 (level 1): mild stage**

• Minor painless skin changes or dermatitis; erythema (skin redness), edema, hyperkeratosis (skin becomes more hard and thick).

• Removal of previous hyperkeratosis prior to initiation of treatment.

• Avoid strenuous exercise and activities that put pressure on your hands and feet for the first 2-4 weeks after treatment.

• Do not wear shoes that are too tight, but wear well-fitting shoes (no sandals, slippers, or high heels).

• Wear cotton socks that fit well and absorb sweat well when you are out and about.

• Wear cotton gloves and socks when sleeping at night.

• Protect your hands by wearing gloves when performing tasks that require hands or when washing dishes or cleaning.

• Not washing your hands too often and avoiding hot water.

• Avoid hand products that contain alcohol.

• Using hand cream and foot cream.

• Apply cushion gel to areas prone to calluses.

• Using a keratolytic urea cream or moisturizer on calluses.

• Water intake of 8-12 glasses (about 200cc water cup) per day.

• Apply a moisturizer to prevent recurrence once the dead skin area is healed.

• Avoid applying ice directly to affected hands and feet.

• Avoid swimming pools and jacuzzi spas.

• Apply an alcohol-free moisturizing cream to prevent your hands and feet from drying out.

• Wear cotton gloves and socks to absorb the moisturizing cream.

• When cutting nails, cut them straight and do not cut them too short.

• Avoid tight-fitting shoes and avoid putting friction or pressure on your limbs.

**Grade-2 (2nd level): moderate stage**

• Skin peeling along with pain, blisters, swelling, bleeding, skin changes such as hyperkeratosis (keratosis, hardening and thickening), functional impairment of daily life.

• Receive regular medical check-ups for local wound treatment and symptom management.

• Apply prescribed painkillers, steroids, antibiotics, and keratolytics well.

• Apply Vaseline to moisturize the skin.

**grade-3 (level 3): severe stage; emergency room visit or outpatient visit or nurse consultation**

• Painful severe skin changes (e.g. peeling, cracking, blistering, hemorrhage, swelling, keratosis); difficulty doing daily life by oneself.

• Gastric cancer: outpatient

• Colorectal cancer: outpatient

• Lung cancer: outpatient

• Breast cancer: outpatient

**4. Changes in fingernails and toenails (falling out, lines, color changes)**

**Grade-1 (level 1): mild stage**

• Do not cut your fingernails and toenails too short.

• Frequently apply lotion or cream to keep your fingernails and toenails from drying out.

• Do not use nail polish as it is irritating.

• Do not use artificial fingernails due to the risk of infection.

• Wear padded shoes to protect your toenails.

• Wear gloves while washing dishes.

**Grade-2 (2nd level): moderate stage**

• Take prescribed antibiotics or apply topical antibiotics properly.

• Avoid actions that put pressure on your nails.

• Cut your toenails short and protect them with bandages.

• Apply cold compresses to the nails.

• When paronychia develops, avoid applying pressure around the nail.

• Do not soak in chemicals or water for a long time.

• Apply moisturizing cream.

• Do not bite your nails or push your nail cuticles.

• Do not cut your toenails too short.

**Grade-3 (level 3): severe stage; emergency room visit or outpatient visit or nurse consultation**

• Medical consultation is required in the following cases:

- If the skin at the root of the nail turns red or is painful and oozes

• An emergency visit is necessary when:

- High fever (38 degrees or more)

- wound infection

- Gastric cancer: emergency room

- Colorectal cancer: outpatient

- Lung cancer: outpatient

- Breast cancer: outpatient

**Ⅵ. Nervous system symptoms**

**1. Peripheral neuropathy**

**Grade-1 (level 1): mild stage**

• It is a mild symptom and does not require special treatment as it recovers over time.

• Primary prevention is not possible as there is no known preventive method or drug.

• If you are taking oxaliplatin injection, avoid cold objects for a few days.

**Grade-2 (2nd level): moderate stage**

• Restrictions in important daily activities (work, school, shopping, housework, etc.) occur.

• Difficulties in everyday life using tools, such as food preparation, require assistance.

• If symptoms get worse and interfere with daily life, you need to consult with your doctor.

If these symptoms occur, damage such as falls or burns may occur. For your safety, please use the following methods.

* Helpful Methods to Relieve Symptoms

• Imaginary therapy with pleasant imagination, relaxation therapy to relax muscles through deep breathing, conversion therapy to turn attention to other places, and cognitive behavioral therapy to manage symptoms by acting with positive thoughts can help relieve symptoms.

• Heat/cold therapy such as a foot bath or hand and foot massage may be helpful if the pain is severe. However, some anticancer drugs prohibit hand and foot massage, so proceed after consulting with the medical staff.

• Rehabilitation treatment can be helpful if peripheral neuropathy is so severe that it is difficult to maintain daily life.

• Exercise helps reduce pain by strengthening muscles and prevents deformities by maintaining muscle and ligament length.

→ If your feet are numb: walking or water rehabilitation

→ In case of numb hands: Actions to improve fine motor skills (picking up go stone, beans, red beans, rice, etc.)

• Desensitization therapy, which gradually stimulates the senses to adapt, helps maintain function and improve walking ability.

→ Sprinkle water on the sore spot with a shower

→ Using silk to stimulate the sore spot

• If possible, walk or do light exercise to maintain strength.

• Wear gloves or shoes to protect your hands and feet when doing household chores like washing dishes or gardening.

• Check your feet daily for bedsores or cuts, as you may not feel your shoes are tight.

• Use thick-handled pots or plastic utensils when cooking.

• If it is difficult to distinguish between cold and hot water, lower the temperature of the water heater and, if possible, ask someone else to check the water temperature.

• Use non-slip mats, shower chairs, and bathtub railings when bathing.

• Brighten up your room or hallway and keep it organized so you don't trip or trip.

• Lighten your stairs and use handrails.

• If buttons or zippers are difficult to handle, wear clothing with elastic bands (elastic pants/skirts, elasticated pants, etc.), or ask an occupational therapist for help with assistive devices.

• If you have a problem with the sense of position of your feet, it may cause problems when you step on the brake pedal of a car, so avoid driving and look at your toes while walking to avoid falling.

• Discuss with your family about any areas you may need help with, such as cooking, cleaning, or tidying up the house.

• Talk to your doctor or nurse and seek help from a physical or occupational therapist.

• Consult your doctor or nurse to prescribe medications to relieve pain, numbness or tingling.

• Talk to the nurse about non-pharmaceutical treatments, such as massage, exercise, acupuncture needles, and topical ointments. It can reduce the discomfort caused by peripheral neuropathy.

**Grade-3 (level 3): severe stage; emergency room visit or outpatient visit or nurse consultation**

• Severe symptoms; difficulty doing daily life by oneself

• Gastric cancer: outpatient

• Colorectal cancer: outpatient

• Lung cancer: outpatient

• Breast cancer: outpatient

**Ⅶ. Psychiatric symptoms**

**1. Cognitive dysfunction, delirium, memory, and concentration problems**

**Grade-1 (level 1): mild stage**

- Efforts are made to help orientation as follows (for guardians).

• Provide adequate lighting for each day and night, and keep a clock and calendar where the patient can see them.

• Frequently and clearly explain where you are, who you are, what you are hospitalized for, and the role of the staff.

• Regular visits from family and friends are helpful.

• Use glasses and hearing aids to maintain sensory function.

• Actively control pain to help walking.

• Provide sufficient fluids and nutrients to avoid dehydration.

• Administer oxygen to prevent hypoxia if breathing difficulties occur.

• Helps smooth bowel movement.

• If you are at high risk of delirium, you must notify your health care provider immediately when symptoms occur.

**Grade-2 (2nd level): moderate stage**

• Control ambient noise and optimize your sleeping environment.

• Care should be taken to prevent complications such as deterioration in motor skills, falls, bedsores, dehydration, and nutritional deficiencies due to delirium.

• Restraints can be used in unavoidable cases, but since physical coercion itself can aggravate delirium, it is applied only in the following cases and for the minimum amount of time.

- If there is a possibility of a fall, self-injury, or other harm

- If symptoms persist despite active efforts such as drug treatment

• Direct treatment by finding the cause is the priority, and drug treatment can be performed to relieve acute symptoms.

• Reduce, discontinue, or change to an alternative drug, if possible.

• Provide objects to help with orientation. (Clocks, calendars, meaningful photos, etc.)

* Guardian Behavior Guidelines

• Empathically ask questions and encourage while understanding the patient's psychological state.

• Keep your view unobstructed and notify the patient before touching or leaving the area.

• Suggest clue words to reinforce awareness of people and places and times.

• Provide clear and concise information, use short words, and speak face-to-face.

• Allow time for answers when questions and decisions need to be made.

• Shift the topic of conversation instead of restricting actions or words.

• No alcohol or unnecessary drugs.

• Create a familiar environment by keeping objects in the same places where they are safe from harm.

• Keep the bed low and the call bell within reach.

**Grade-3 (level 3): severe stage; emergency room visit or outpatient visit or nurse consultation**

• Severely impaired concentration or level of consciousness; difficulty doing daily life by oneself

• Stomach cancer: not applicable

• Colorectal cancer: not applicable

• Lung cancer: emergency Room

• Breast cancer: not applicable

**2. Insomnia (Sleep Disorder)**

**Grade-1 (level 1): mild stage**

• Some medications, such as steroids, can cause insomnia, so adjust the intake time after consulting with your doctor.

• Actively manage insomnia as it can sometimes be a sign of anxiety or depression.

• Stress reduction methods, relaxation, imagery, progressive muscle relaxation, expressive group therapy, writing, massage, exercise, meditation, yoga, music therapy, biofeedback, cognitive-behavioral therapy, acupuncture, aromatherapy, therapeutic touch, spirit therapy, etc., can help.

* Sleep Habit Tips

• Try to go to bed and wake up at the same time each day.

• Stay active and exercise during the day long enough to feel tired and take short naps in the afternoon, not too late.

• Avoid caffeine, cigarettes, stimulating meals, and excessive alcohol late in the day.

• Avoid exercising or eating two hours before going to bed, especially drinking water, and urinate just before bed.

• Try taking a bath, aromatherapy, foot massage, reading a book, listening to soothing music or an audio book, or drinking a warm milk drink before bed.

• The sleeping environment is a separate room, dark, quiet and not too hot, and the bedding is clean and neat without wrinkles.

• If you have difficulty falling asleep within 15 to 20 minutes, get up, move to another room, and go back to your bedroom when you feel sleepy again. Repeat as many times as necessary.

• Set an alarm to wake up at the same time every morning regardless of how long you sleep.

• In bed, only sleep and sex life. (Don't watch TV in bed)

• Work to address any health conditions that are interfering with sleep. For example, take an antiemetic or pain reliever to relieve symptoms and thoroughly manage heartburn and high blood sugar.

• Refrain from using your smartphone before sleeping.

**Grade-2 (2nd level): moderate stage**

• If, despite your best efforts, your sleep disturbance causes discomfort in your daily life, you should consult with your healthcare provider.

**Grade-3 (level 3): severe stage; emergency room visit or outpatient visit or nurse consultation**

• Difficulty falling asleep, waking up frequently, or waking up early

• Stomach cancer: not applicable

• Colorectal cancer: not applicable

• Lung cancer: outpatient

• Breast cancer: outpatient

**3. Fatigue, fatigue or lack of energy**

**Grade-1 (level 1): mild stage**

- Fatigue is reduced by rest

* How to help with fatigue in everyday life

• Provide a balanced amount of rest between physical activities.

• Maintain a normal life, but take a break as soon as you feel tired.

• Expose to the natural environment to recover concentration.

• Do enjoyable activities such as listening to music, watching TV, or participating in religious or social activities.

• Get help from people around you in your daily life, and keep things you use all the time in an easy-to-reach place so you don't waste energy.

• Physical activity, such as a light walk, improves your appetite and helps with fatigue. However, it is not recommended to exercise in the evening.

* A diet conducive to fatigue

• Eat a balanced diet that includes enough fluids, calories, protein, carbohydrates, fats, vitamins and minerals.

• Limit alcohol and caffeinated foods, especially in the late afternoon and evening.

* Improve sleep quality

• Limit fluids just before going to bed and maintain a quiet, comfortable environment before going to bed.

• Go to bed at a similar time each day and wake up at a regular time each morning.

• Avoid naps or limit them to 30 minutes.

**Grade-2 (2nd level): moderate stage**

- Fatigue does not decrease even when resting, daily life is limited

• Energy Conservation Method: This is a method of conserving and conserving energy by adjusting daily life to reduce fatigue. In particular, cancer-related fatigue tends to be worse in the afternoon, so it's good to plan and do things that consume a lot of energy in the morning.

• Plan systematically ahead of time what needs to be done.

• Prioritize to reduce or eliminate less important tasks.

• Delegate tasks or use energy saving devices.

• We work at times when we are at our peak energy and focus.

• Work Pace: Adjust the pace and order of work so that activities and breaks can be repeated.

• Posture: If possible, sit down frequently even during activities.

• Wear loose-fitting clothing that is easy to put on and take off.

• If you feel tired from eating or preparing food, have others serve or prepare the food for you.

• Use food that does not require special preparation, such as canned or frozen food, or order food from outside.

**grade-3 (level 3): severe stage; Emergency room visit or outpatient visit or nurse consultation**

• Fatigue not relieved by rest, difficulty in performing daily activities by oneself.

• Gastric cancer: outpatient

• Colorectal cancer: nurse consultation

• Lung cancer: nurse consultation

• Breast cancer: nurse consultation

**4. Anxiety**

**Grade-1 (level 1): mild stage**

• Talk to your doctor to correct misconceptions about your illness

• Receive explanations of unrealistic anxiety about possible future course of illness.

• Fully listen to explanations before examination or treatment.

• Eliminate factors that exacerbate anxiety (lack of sleep, poor pain control, high doses of caffeine, nicotine withdrawal symptoms, etc.).

• Talk openly about your feelings to family and friends. Fear, sadness, loneliness, any emotion is fine.

• Talk about your struggles and try to find and understand what you can do for each other.

• Try to have a conversation, but don't force it.

• Find a person, minister, or group who can provide an interview or emotional empathy.

• Don't blame yourself or others when you feel anxious or afraid. Instead, look for the cause of your anxiety and fear and talk about it.

• Try deep breathing or relaxation several times a day. When trying to breathe deeply, try to make the exhalation longer than the inhalation. When things aren't going well, just focusing on your breathing can help.

• How to relax: Close your eyes, take a deep breath, focus on each part of your body, and relax each muscle one by one. Imagine being in a pleasant place, such as an early morning beach or arboretum, when your entire body is relaxed enough.

• Quiet music, art, reading or watching movies can reduce anxiety.

**Grade-2 (2nd level): moderate stage**

• After consultation with your doctor or psychiatrist, you can receive a prescription for appropriate medication.

• If you feel anxiety that makes your daily life difficult or does not get better despite your efforts, taking medication can be effective.

• Some medications can make anxiety worse. If your anxiety gets worse after a new medication is added, tell your doctor and discuss it.

**Grade-3 (level 3): severe stage; emergency room visit or outpatient visit or nurse consultation**

• Severe symptoms; difficulty performing daily activities on their own; hospitalization required.

• Stomach cancer: not applicable

• Colorectal cancer: not applicable

• Lung cancer: not applicable

• Breast cancer: outpatient

**5. Depression**

**Grade-1 (level 1): mild stage**

• Depression is common in cancer patients. This doesn't mean you're weak. Talk to your nurse and doctor about your feelings.

• Depression is not uncommon in cancer patients, and managing depressive symptoms can improve cancer treatment outcomes by improving quality of life, improving communication between patients and medical staff, and improving treatment adherence.

• Talk openly about your feelings to family and friends. Fear, sadness, loneliness, any emotion is fine.

• Get plenty of rest and exercise regularly. Light physical activity increases the energy of the mind and body.

• Make a list of the medications you take and show them to your nurse or doctor. Some drugs may need to be stopped.

• Check with your nurse or doctor about cancer and side effects from treatment. And learn things to help you manage side effects.

• Ask your nurse or doctor for help managing side effects.

• Eat a balanced diet and don't lose weight. Many nutrients in food are very important for maintaining good health. If you have difficulty eating, ask your nurse or nutritionist for help.

• Avoid drinking alcohol. Alcohol makes you depressed.

**Grade-2 (2nd level): moderate grade**

• During the course of cancer treatment, it is recommended not to take it for granted or hide the problem for fear of double stigma, but to receive a prescription for appropriate medication after consulting with your doctor or psychiatrist.

• In particular, if you suffer from depression that makes daily life difficult or your depression does not recover despite your efforts, taking medication together can be effective.

• If medications for depression are prescribed, take them as directed. If you have any side effects, tell your nurse or doctor.

• If you have suicidal thoughts, actively inform others about your condition and seek help from a mental health professional.

**Grade-3 (level 3): severe stage; emergency room visit or outpatient visit or nurse consultation**

• severe depressive symptoms; difficulty doing daily life by oneself.

• Stomach cancer: not applicable

• Colorectal cancer: not applicable

• Lung cancer: not applicable

• Breast cancer: outpatient

**Ⅷ. Pain**

**1. Pain**

**Grade-1 (level 1): mild stage**

- minor pain

• Medication management

• Unless prohibited, non-narcotic pain relievers may be prescribed and taken regularly or as needed.

• Non-drug management

• Relaxation, walking, relaxation, relaxation, meditation, yoga, acupressure, deep breathing, and more.

**Grade-2 (2nd level): moderate stage**

- Moderate pain, moderate pain that interferes with important daily activities (work, school, shopping, housework).

• Management method

- Take oral narcotic analgesics regularly as prescribed.

- In case of breakthrough pain of 4 points or more, take a short-acting analgesic.

- Keep a pain diary to record the medications you take and pain patterns so that you can discuss them with the medical staff when you visit the hospital.

• Medical consultation is required in the following cases.

- If the pain is not controlled by taking painkillers

- If the side effects of painkillers are unbearable

**Grade-3 (level 3): severe stage; emergency room visit or outpatient visit or nurse consultation**

• Pain score of 7-10, high degree of pain, severe pain that makes it difficult to carry out daily activities (dressing alone, going to the bathroom).

• Gastric cancer: outpatient

• Colorectal cancer: outpatient

• Lung cancer: outpatient

• Breast cancer: outpatient

**Ⅸ. Urinary system, gynecological symptoms and sexual function**

**1. Hemorrhagic cystitis, hematuria**

**Grade-1 (level 1): mild stage**

- Mild symptoms, but no treatment required.

• Drink at least 2L of water per day unless specifically restricted by your doctor.

• While receiving anticancer drugs, drink plenty of water, juice, or tea after taking them and urinate frequently.

• If you want to urinate, don't hold back and go to the bathroom immediately.

• Do pelvic Kegel exercises.

• Reduce drinking water after dinner.

• Avoid eating foods that irritate the bladder (cola, spicy food, coffee, alcohol).

• Avoid taking a bath.

**Grade-2 (2nd level): moderate stage**

- Moderate symptoms requiring treatment. There are limitations in daily life.

* Management method

• fluid therapy

• If you need a medical consultation

• If your urine changes color or bleeds.

• Pain in the back or lower abdomen when urinating

• Urine smells bad and is cloudy in color.

• When you feel the urge again soon after urinating

• Urinating too often than usual

• When urine does not come out even if you exert force

• When you have a fever of 38 degrees or higher or chills

**Grade-3 (level 3): Severe stage**

• Severe bleeding makes it difficult to do daily activities on your own

• An emergency visit is needed if:

- When the blood doesn't stop

- High fever (more than 38 degrees)

**2. Menopausal Syndrome**

**Grade-1 (level 1): mild stage**

• Facial flushing

• As body fat increases, symptoms tend to get worse, so regular exercise and proper weight maintenance are necessary.

• Avoid hot and humid environments and confined spaces.

• Avoid stress, smoking, caffeine, alcohol, hot drinks, spicy foods, and using a hair dryer.

• Stress management such as meditation, yoga, and biofeedback may reduce the intensity of hot flashes.

• Wear several layers of thin clothing and wear non-constricting cotton or natural fiber clothing.

• Drink cool water and use an ice pack if needed.

• Use a fan or open a window to circulate the air, keeping the room cool and at a reasonable level.

• Vaginal dryness, dyspareunia management

- It may help to have regular sex to improve the flexibility of the vagina.

- Regular Kegel exercises are recommended to improve the elasticity of the muscles and blood vessels of the vaginal tissue.

- Avoid perfumes, antihistamines, soaps, deodorants, powders, spermicides, panty liners, and tight-fitting clothes.

- Use a water-soluble vaginal moisturizing lubricant during sexual intercourse. Do not use those that contain fragrance or alcohol.

- If symptoms are severe or worsen, oil-based vaginal moisturizing lubricants can be used.

- Estrogen vaginal rings, vaginal tablets, and vaginal creams can be used, but the safety of intravaginal estrogen use in women with a history of breast cancer is controversial.

• Inform your healthcare provider if you experience vaginal itching or pain on a regular basis, or if vaginal discharge has a foul odor. You do not necessarily need a tertiary hospital treatment, so it is recommended that you seek treatment at a nearby gynecological clinic.

Grade-2 (2nd level): moderate stage

• If you feel that your symptoms are intolerable or that they are affecting your health, tell your health care team. There are medications and prescriptions available for your needs.

• Management method

- Fluid treatment

• Medical consultation is required in the following cases:

- Change in urine color or blood

- Pain in the back or lower abdomen when urinating

- When the urine smells bad and the color is cloudy

- When you feel the urge to urinate again soon after urinating

- Urinating more often than usual

- When urine does not come out even if you exert force

- When you have a fever of 38 degrees or higher or chills

**Grade-3 (level 3): severe stage; emergency room visit or outpatient visit or nurse consultation**

• Severe discomfort or pain during vaginal passage (discomfort or pain that is not relieved by vaginal lubricants or estrogen).

• Stomach cancer: not applicable

• Colorectal cancer: not applicable

• Lung cancer: not applicable

• Breast cancer: nurse consultation

**3. Sexuality and fertility problems**

**Grade-1 (level 1): mild stage**

• Have an honest conversation with your partner about your lack of sexual interest.

• Talk to your partner about any concerns or struggles with your sex life, and talk about and try other coping strategies other than intercourse that can help you feel sexually fulfilled. (Explore together ways to increase intimacy, such as touching and caressing other than the genitals, such as kissing or hugging.)

• Actively manage the side effects of chemotherapy and medications you are taking so that they do not interfere with your sex life.

• Take pain relievers or antiemetics on time.

• If you have a stoma or stoma, use a concealable belly band or underwear and be careful not to rub the pocket.

• Try to conserve energy to reduce fatigue during sex.

• Create a new sexual environment by using candlelight, music, or suggestive clothing.

• By tightening and relaxing the vaginal muscles, women's Kegel exercises can increase the elasticity of the vaginal muscles and improve sexual satisfaction.

• Try changing positions. (Man behind woman, lying side by side, woman on top, raising the head of the bed, etc.)

**Grade-2 (2nd level): moderate stage**

• For women, if you use vaginal lubricant and nuclear stimulator 3 times a week to manage vaginal dryness, you can have more comfortable sex.

• You can talk to your health care provider about hormone replacement therapy or vaginal reconstruction. However, those who have been treated for breast cancer are difficult to use hormone replacement therapy.

• For men, there are FDA-approved treatments for erectile dysfunction. Common side effects include headache, dizziness, facial flushing, indigestion, nasal congestion, etc. The frequency of side effects varies depending on the existing health condition, and in rare cases, unexpected serious side effects may occur.

• Seek help from a urologist as non-drug options may also be available.

**Grade-3 (level 3): Not applicable.**

**Ⅹ. Hematological abnormalities**

**1. Anemia**

**Grade-1 (level 1): mild stage**

• Hemoglobin (Hgb) <10.0 g/dL

• Become familiar with anemia-related symptoms, write them down in a journal, and report all related symptoms to your doctor at the time of treatment. (severe fatigue or weakness, shortness of breath, pale skin including pale lips, gums, eyelids, nail roots, palms, etc.; fast heart rate, etc.)

• Do not overdo your daily activities.

**Grade-2 (2nd level): moderate stage**

• Hemoglobin (Hgb) <10.0 - 8.0 g/dL

* Management method

• Activities such as driving, babysitting, and going out require attention when dizzy.

• Rise slowly from a lying or sitting position.

• Get plenty of rest.

• Medical consultation is required in the following cases:

- Severe fatigue or weakness, shortness of breath, fast heart rate, etc.

- Urinating more often than usual

**Grade-3 (level 3): Severe stage**

• Hemoglobin (Hgb) <8.0 g/dL

• An emergency visit is needed if:

- Sudden onset of very severe fatigue

- Chest pain

- Tachycardia

- Shortness of breath

- Rapid bleeding

**2. Thrombocytopenia**

**Grade-1 (level 1): mild stage**

• < 75,000/mm3;

* Preventive management

• Be careful not to scratch, tear or injure your skin with sharp objects.

• Use an electric razor.

• Be careful when trimming your toenails.

• Use a soft toothbrush.

• Do not use dental floss or toothpicks.

• Maintain normal bowel movements and avoid constipation.

• Do not insert suppositories or give enemas.

• Wear non-slip shoes.

**Grade-2 (2nd level): moderate stage**

• <75,000 - 50,000/mm3

* Management method

• If bleeding occurs, apply direct pressure to the bleeding area for at least 10 minutes.

• If there is bleeding in a limb, elevate the bleeding site above the heart.

• If nosebleeds occur, cover them with gauze or tissue.

• If you have abnormal vaginal bleeding, check how many pads you need to use.

**Grade-3 (level 3): severe stage**

• <50,000 - 25,000/mm3

• Medical consultation is required in the following cases.

- Persistent nosebleeds

- black stools

- severe headache

- Feces mixed with blood

- Bleeding when coughing

- blood in urine

- Sudden eye redness and changes in vision

- Red spots on any part of the body

- Bleeding at the site of central venous catheter insertion

- Abnormal vaginal bleeding (if not regular menstrual period)

Bleeding on the gums, lips or small blood blisters in the mouth

• An emergency visit is needed if:

- Bleeding that continues despite pressure hemostasis for at least 10 minutes

- Shortness of breath and difficulty breathing

- If there is a change in consciousness

**3. 호중구감소증**

**Grade-1 (level 1): mild stage**

• Wash your hands thoroughly before eating, after going out, and after going to the bathroom.

• Brush your teeth frequently with a soft toothbrush.

• Avoid contact with people with contagious diseases.

• Wash fruits and vegetables carefully, and avoid eating raw meat, chicken, fish, or eggs.

• Do not pick up animal droppings or exchange cat litter yourself.

**Grade-2 (2nd level): moderate stage**

• If you feel unwell, check your temperature.

• Look for signs of infection.

**Grade-3 (level 3): severe stage**

• If the neutrophil count is less than 1000/mm3 and the body temperature is 38.3 degrees or higher or a fever of 38.0 degrees or higher lasts for more than 1 hour.

• An emergency visit is needed if:

- Body temperature of 38.3 degrees or higher or a fever of 38.0 degrees or higher for more than 1 hour

- If there is a change in the state of consciousness

- If accompanied by severe headache or shortness of breath

**ⅩⅠ. Other symptoms**

**1. Bleeding**

**Grade-1 (level 1): mild stage**

It is most important to prevent bleeding through daily management

• skin

- Clean around the perineum daily.

- Apply lotion to prevent weakening and drying of the skin.

- Be careful not to get cut or abrased by sharp objects

- Shave using an electric razor.

- Cut fingernails and toenails short and trim the ends well.

• Be careful not to fall or fall from a height.

• It is recommended not to engage in sports where there is a possibility of collision with others (such as soccer, basketball, boxing, and skating).

• Oral care

- Brush your teeth after eating and before going to bed.

- Use a soft-bristled toothbrush.

- If your platelet count is low, use a cotton swab or mouth sponge instead of a toothbrush.

- Alcohol causes dry mouth, so use a mouthwash that does not contain alcohol.

• digestive system

- Maintain normal bowel function and avoid overwork and constipation.

• You should take stool laxatives (medicines that prevent or treat constipation) prescribed by your doctor.

- Eat right and exercise.

- Do not take a temperature rectal using a rectal thermometer.

• Other management

- Do not extract teeth during chemotherapy.

**Grade-2 (2nd level): moderate grade**

• If there is bleeding, do not panic and sit or lie down to rest.

• For external bleeding such as cuts, abrasions or nosebleeds, apply pressure for at least 10 to 15 minutes to stop the bleeding.

• If bleeding is from an arm or leg, keep the arm or leg elevated above the heart (chest level).

• Applying an ice pack to the bleeding area for 5 to 10 minutes may help stop the bleeding.

• Frequent oral care is required when mouth bleeding occurs.

• If there is blood in the vomit, take an anti-emetic, antacid, or stomach acid-reducing medicine prescribed by your doctor. Spicy and acidic foods and consumption of caffeine should be avoided.

• Drink plenty of water or soft drinks when you have bloody urine (hematuria).

• Tell your doctor the color and amount of your urine.

• If you have heavy or unusual vaginal bleeding, tell your doctor the amount and pattern of vaginal bleeding and the size of the clot. The number of sanitary napkins used should be reported to the doctor. Tampons are not used.

**Grade-3 (level 3): severe stage; emergency room visit or outpatient visit or nurse consultation**

• Uncontrolled bleeding (requires blood transfusion; requires invasive procedures).

• Stomach cancer: emergency room

• Colorectal cancer: emergency room

• Lung cancer: emergency Room

• Breast cancer: emergency room

**2. Injection site pain and swelling (extravasation)**

**Grade-1 (level 1): mild stage**

• If the blood vessels are not good, a special tube is used to inject the drug.

• Avoid injections in the arm where the mastectomy was performed, and in the lower extremity where there is a risk of blood clots.

• Be aware of any training you have received from your healthcare provider about the risk of extravasation.

• Take the chemotherapy in a comfortable position.

• Express discomfort at the injection site, feeling of drug leakage, pain, and burning without hesitation.

**Grade-2 (2nd level): moderate stage**

• Cold compress

- Causes vasoconstriction, minimizing the spread of the drug to other tissues, and reducing local inflammation and pain.

- Apply 15 to 20 minutes per session 4 to 6 times a day for more than 1 day.

- Applied drugs: Doxorubicin, daunorubicin, epirubicin, etc.

• Warm compresses

- It expands the blood vessels in the extravasation area and increases the blood flow to increase the dispersion and absorption of the medicinal solution, helping the leaked medicinal solution to spread quickly from the extravasation area.

- Apply 20 to 30 minutes per session 4 to 6 times a day and apply for more than 1 day.

- Applicable drugs: Vincristine, vinblastine, paclitaxel, etc.

**Grade-3 (level 3): severe stage; emergency room visit or outpatient visit or nurse consultation**

• Ulcers or necrosis, with severe tissue damage (requires surgical intervention)

• Stomach cancer: not applicable

• Colorectal cancer: emergency room

• Lung cancer: not applicable

• Breast cancer: emergency room

**3. Fever**

**Grade-1 (level 1): mild stage**

• Wash your hands often with soap and water.

• Maintain good oral hygiene and personal hygiene.

• Keeps wounds clean and dry.

• Avoid crowded places.

• Hydration is recommended.

• Wash raw fruits and vegetables well and cook meat and fish at the right temperature.

• Avoid contact with people who are ill.

**Grade-2 (2nd level): moderate stage**

• Consultation with medical staff is required in the following cases:

- When body temperature is over 38.5 degrees

- When the fever lasts more than 72 hours

- Severe other systemic symptoms, such as a stiff neck, colored phlegm, or pain when urinating

- Pain, redness, and swelling at the catheter insertion site

- New symptoms or signs

- Persistent or recurring fever 48 hours after initiation of antibiotic treatment

- Unable to continue taking prescribed antibiotics

• An emergency visit is necessary if:

- When body temperature is above 38 degrees

- Stiff throat or colored phlegm

- Shortness of breath

- Seizures or confusion

- Severe vomiting or diarrhea)

- Chest pain or shortness of breath

- Abnormal vital signs: hypotension, tachycardia

**Grade-3 (level 3): severe stage; emergency room visit or outpatient visit or nurse consultation**

• A fever of 39 degrees or higher

• Stomach cancer: emergency room

• Colorectal cancer: emergency room

• Lung cancer: emergency room

• Breast cancer: emergency room
